# Neuropeptide substance P attenuates colitis by suppressing inflammation and ferroptosis via the cGAS-STING signaling pathway

**DOI:** 10.7150/ijbs.94548

**Published:** 2024-04-22

**Authors:** Jing Lan, Ziteng Deng, Qiuzhen Wang, Dan Li, Kai Fan, Jianyu Chang, Yunfei Ma

**Affiliations:** State Key Laboratory of Veterinary Public Health and Safety, College of Veterinary Medicine, China Agricultural University, Beijing, China.

**Keywords:** Substance P, Colitis, mtDNA, cGAS-STING, Inflammation, Ferroptosis

## Abstract

Neuropeptide substance P (SP) belongs to a family of bioactive peptides and regulates many human diseases. This study aims to investigate the role and underlying mechanisms of SP in colitis. Here, activated SP-positive neurons and increased SP expression were observed in dextran sodium sulfate (DSS)-induced colitis lesions in mice. Administration of exogenous SP efficiently ameliorated the clinical symptoms, impaired intestinal barrier function, and inflammatory response. Mechanistically, SP protected mitochondria from damage caused by DSS or TNF-α exposure, preventing mitochondrial DNA (mtDNA) leakage into the cytoplasm, thereby inhibiting the cyclic GMP-AMP synthase-stimulator of interferon genes (cGAS-STING) pathway. SP can also directly prevent STING phosphorylation through the neurokinin-1 receptor (NK1R), thereby inhibiting the activation of the TBK1-IRF3 signaling pathway. Further studies revealed that SP alleviated the DSS or TNF-α-induced ferroptosis process, which was associated with repressing the cGAS-STING signaling pathway. Notably, we identified that the NK1R inhibition reversed the effects of SP on inflammation and ferroptosis via the cGAS-STING pathway. Collectively, we unveil that SP attenuates inflammation and ferroptosis via suppressing the mtDNA-cGAS-STING or directly acting on the STING pathway, contributing to improving colitis in an NK1R-dependent manner. These findings provide a novel mechanism of SP regulating ulcerative colitis (UC) disease.

## Introduction

The pathogenesis of inflammatory bowel disease (IBD), including Crohn's disease and ulcerative colitis (UC), is still not fully understood. UC carries a higher risk of developing colon cancer if not effectively treated, ultimately contributing to severe threats to life 1, 2. According to current therapies, widely used clinical drugs include monoclonal antibodies, 5-aminosalicylic acid, and so on. However, these treatments may have long-term side effects or only relieve the pathologic condition of patients in a short time 3. Therefore, it is very urgent to elucidate the pathological mechanisms and seek novel therapeutic methods for UC. Importantly, in recent years, researchers have noticed that the enteric nervous system (ENS) functions as a control center for managing gastrointestinal physiology 4, 5. As a result, dysfunction of the ENS impairs gut homeostasis, leading to gastrointestinal and extragastrointestinal diseases. The enteric neurons in the myenteric and submucosal ganglia play a crucial role by encoding distinct neurochemicals with specific functions 6. Neuropeptide substance P (SP) is an 11-amino acid peptide and was first discovered in the equine brain and intestine in 1973 7. This neuropeptide is primarily distributed in the central and peripheral nervous systems and is also secreted by non-neural cells, such as endothelial cells, epithelial cells, and immune cells 7-10. SP has been shown to exert biological impacts through G protein-coupled receptors (GPCRs), particularly neurokinin receptors (NKRs), including neurokinin 1 receptor (NK1R), NK2R, and NK3R 11, 12. In addition, it has been reported that NK1R manifests the highest affinity for SP and participates in a multitude of biological processes, such as pain neurotransmission, inflammatory regulation, proliferation, and anti-apoptosis 13-16. Although previous studies have explored the role of SP in mediating inflammation-related diseases in the respiratory, gastrointestinal, and musculoskeletal systems 17-21, the protective effects of SP have not been completely clarified. Notably, recent studies have reported that neuropeptide SP exerts significant effects in protecting against cardiovascular dysfunction, skin ulcers, and intestinal disorders 22-24. Emerging evidence has demonstrated that SP released from transient receptor potential vanilloid subtype 1+ (TRPV1+) neurons in response to microbial dysbiosis could improve the development of colitis 24. However, the underlying action mechanism of SP/NK1R signaling in intestinal inflammatory disease has not been elucidated.

In recent years, more and more researchers have paid attention to the emerging role of the cyclic GMP-AMP synthase (cGAS) stimulator of the interferon genes (STING) pathway. The cGAS-STING pathway is a critical signaling cascade of the innate immune system that is activated upon sensing bacterial DNA or self-DNA, initiating downstream inflammatory factors in the pathological progression of various diseases 25. The mitochondrial DNA (mtDNA) is released into the cytosol after cellular damage and stress and activates STING signaling via cGAS, resulting in systemic immune response and organ disorder 26. In particular, the latest studies have reported the involvement of the cGAS-STING signaling pathway in colitis and CLP-induced lethal sepsis 27, 28. These compelling findings underscore the significance of cGAS-STING signaling as a promising therapeutic target for developing safe and efficacious drugs for UC treatment. Previous studies have discovered that SP couples to PI-PLC through the NK1 receptor in the rat anterior pituitary by binding with GTP, which serves as the substrate for cGAS catalysis of cGAMP formation 29, 30. This suggests that SP may affect the activation of the cGAS-STING signaling pathway. Nevertheless, the role of SP in modulating STING transcription in the context of UC pathogenesis remains unknown. On the other hand, ferroptosis, a unique form of non-apoptotic cell death, can be initiated by the accumulation of iron and production of reactive oxygen species (ROS) from lipid peroxidation 31. Several studies have identified ferroptosis as an important contributor to the occurrence and progression of various diseases and inhibiting ferroptosis may lessen inflammatory response and intestinal epithelial cells (IECs) injury in IBD 32-34. However, it remains unclear whether the intestinal cGAS-STING pathway is involved in IEC ferroptosis during colitis and how SP/NK1R signaling affects it in maintaining epithelial homeostasis.

The present study aimed to investigate the signaling mechanisms by which SP modulates intestinal barrier function in colitis. We found that the number of activated SP-positive neurons and the level of SP were significantly increased in a mouse model of dextran sodium sulfate (DSS)-induced colitis. SP could ameliorate colitis and intestinal epithelium disruption in DSS-induced mice, and the *in vitro* inflammation model of tumor necrosis factor-alpha (TNF-α)-stimulated human colon carcinoma cell line (Caco-2), which was dependent on its specific receptor NK1R. Importantly, we, for the first time, explored the underlying mechanisms and demonstrated that SP inhibited mtDNA-cGAS-STING or directly suppressed the STING pathway to attenuate inflammation and ferroptosis, eventually mitigating the DSS-induced colitis. This study is expected to provide a novel therapeutic basis for SP as a promising intervention to control colitis progression.

## Materials and Methods

### Animals

Forty-eight 6- to 8-week-old male C57/6N mice (20-22 g) were obtained from Beijing Vital River Laboratory Animal Technology Co., Ltd., Beijing, China, and housed (5 mice per cage) in the animal center. Mice were maintained under a 12-h light/12-h dark cycle with access to water and a standard chow diet in specific pathogen-free conditions. The protocol in our study involving experimental mice was approved by the Committee for the Care and Use of Experimental Animals, China Agricultural University (AW92303202-2-1). All animals were treated humanely, and all animal procedures met the relevant legal requirements.

### Mouse colitis model and experimental design

DSS (36000-50,000 Da molecular weight) was purchased from Yeasen (Shanghai, China). The DSS-induced colitis mouse model was established according to our previous laboratory study 35. SP (≥98%) was supplied by MedChemExpress (Shanghai, China), and its structure is shown in Figure 2A. The mice were randomly divided into four groups of 12 mice per group: 1) The control (CON) group was given normal water and intravenously injected with vehicle sterile saline daily for 6 d; 2) The DSS group was provided free access to 4% DSS, and intravenously injected with vehicle sterile saline daily for 6 d; 3) The DSS + SP group was administered 4% DSS and intravenously injected with SP (5 nmol/kg/day in sterile saline) daily for 6 d, and the dose of SP was determined according to a previous study 36; 4) The SP group was given normal water and intravenously injected with SP (5 nmol/kg/day in sterile saline) daily for 6 d. All the mice were weighed every day. The body weight loss and disease activity index (DAI) were monitored and recorded. The mice were anesthetized at the end of the protocol, and blood samples were taken from the mouse eyeball. Then, animals were sacrificed, and their colon tissue length and spleens were measured and weighed separately. The serum was isolated by quickly centrifuging at 3000 rpm for 15 min and then stored at -80 °C. A portion of colon tissue was fixed in 4% paraformaldehyde for histopathological examination, and the remaining portion was immediately stored at -80 °C for further experiments.

### Immunohistochemical staining

Immunohistochemical staining was carried out following a previously published method 35. Sliced colon tissue (4 μm) was embedded in paraffin and dewaxed and hydrated with dimethyl-benzene and alcohol, respectively, by the avidin-biotin complex (ABC) Peroxidase Staining Kit (Thermo Scientific, WA, USA). The samples were permeabilized with Triton-X 100 for 15 mins. Next, the sections were placed in citrate buffer (pH = 6.0), microwaved for antigen retrieval, and blocked in 3% H_2_O_2_ for 30 mins and donkey serum for 1 h. Finally, the sections were incubated at 4 °C overnight with the following corresponding primary antibodies: SP (abs137008, 1:100, Absin, Shanghai, China), proliferating cell nuclear antigen (PCNA, 60097-1, 1:500, Proteintech, Wuhan, China), zona occludens 1 (ZO-1, ab59760, 1:100, Abcam, Cambridge, MA, USA), STING (NBP2-24683, 1:200, NOVUS, Missouri, USA) and NK1R (DF7438, 1:200, Affinity, Michigan, USA). The sections were incubated with biotin-labeled horseradish peroxidase. After that, the tissues were stained using 3,3′-diamino-benzidine-tetrahydrochloride (DAB). Images were obtained by a microscope (Thermo Fisher Scientific, WA, USA).

### Hematoxylin and eosin (H&E) staining

In brief, after being fixed in 4% paraformaldehyde, samples were embedded in paraffin and sliced into sections (4 μm). The tissue sections were deparaffinized and hydrated through a series of gradient alcohols. Then, the sections were stained with hematoxylin and eosin. After washing in distilled water, the slide was dehydrated in alcohol and placed in a transparent solution of xylene. The histological changes were captured using an optical microscope (Olympus, Tokyo, Japan). At least 20 randomly selected fields were assessed under × 400 magnification, and data were averaged for each colon (n = 8). The histological scores were determined by researcher blinding for the experimental group, as previously reported 37.

### Alcian blue periodic acid Schiff (AB-PAS) staining

The mucus secretion capacity of the mice was analyzed by AB-PAS Stain kit (Beijing Solarbio Science & Technology Co., Ltd.) following the manufacturer's guidelines. After dewaxing, the tissue sections were stained with Alcian blue dye solution and then washed in running water. Second, the sections were stained with 0.5% periodic acid solution for 15 mins and washed twice with distilled water. Third, the tissues were soaked in Schiff reagent under the dark conditions for 30 mins and then rinsed for 5 mins. The slides were dehydrated with gradient alcohol and cleared with xylene to be transparent. The sections were observed for the mucus layer and goblet cells under an optical microscope (Nikon Italia, Italy). At least 20 randomly selected fields were assessed to quantify goblet cells under × 400 magnification, and data were averaged for each colon (n = 8).

### Transmission electron microscopy (TEM)

TEM was conducted based on previously described approaches 38. Briefly, colonic segments were cut into 0.1 cm × 0.1 cm × 0.1 cm samples and instantly fixed with 2.5% glutaraldehyde at 4 °C for 4 h. Then, the samples were then dehydrated in ethanol and propylene oxide and embedded in epoxy resin for cutting into ultrathin sections. The sections were stained with uranyl acetate and lead citrate, followed by photography under a TEM-1400 Plus electron microscope (JEOL JEM-1400 Plus, Tokyo, Japan).

### Cytokine array

Total protein was extracted from colonic tissues with RIPA buffer, and the concentration was tested using a BCA kit. The concentrations of 23 cytokines, including interleukin-3 (IL-3), interleukin-1beta (IL-1β), TNF-α, interleukin-9 (IL-9), interleukin-4 (IL-4), monocyte chemoattractant protein-1 (MCP-1), interleukin-5 (IL-5), interferon-gamma (IFN-γ), interleukin-2 (IL-2), macrophage inhibitory protein-1 alpha (MIP-1α), interleukin-13 (IL-13), interleukin-6 (IL-6), interleukin-1alpha (IL-1α), granulocyte colony-stimulating factor (G-CSF), eotaxin-CCL11 (Eotaxin), macrophage inhibitory protein-1 beta (MIP-1β), interleukin 12 p40 (IL-12P40), RANTES, interleukin-10 (IL-10), keratinocyte chemoattractant (KC), interleukin-17 (IL-17A), granulocyte macrophage-colony stimulating factor (GM-CSF) and interleukin 12 p70 (IL-12P70), were assayed in mouse colon tissue and serum samples using Luminex multiplex bead array assays with LabEx (Shanghai, China).

### Assessment of SOD, GSH-Px, T-AOC, and MDA levels

The collected colon samples in each group were homogenized with PBS and then centrifuged (6000 rpm, 10 mins) at 4 °C to obtain supernatants for analysis. The glutathione peroxidase (GSH-Px), total antioxidant capacity (T-AOC), superoxide dismutase (SOD) and malondialdehyde (MDA) levels were detected by commercially available kits according to the manufacturer's instructions (NJJCBIO, Nanjing, China). For antioxidant enzyme assays, the GSH-Px, T-AOC, SOD and MDA contents were determined at 550, 412, 520, and 532 nm, respectively, by a microplate reader (Bio-Rad, Germany).

### Cell culture and treatment

Caco-2 cells (ATCC, Manassas, Virginia, USA) were cultured in DMEM supplemented with 20% FBS and 1% penicillin-streptomycin in a 37 °C humidified incubator with 5% CO_2_. Caco-2 cells were dissociated and seeded in cell plates when grown to 80-90% confluence, and then they could be used for subsequent experiments. TNF-α was used to stimulate Caco-2 cells to imitate the inflammation model *in vitro,* as previously reported 39. Caco-2 cells were treated with SP for 24 h at concentrations of 25, 50, and 100 nmol. Caco-2 cells were treated with an inhibitor of NK1R (L-732138) at 40, 60, and 80 nmol for 2 h prior to SP treatment. DMXAA and SR717, two STING agonists, treated Caco-2 cells at concentrations of 80 μg/mL and 4 μM for 24 h, respectively.

### Cell Counting Kit-8 (CCK8) assay

Cell viability was evaluated by Cell Counting Kit-8 from MedChemExpress (Shanghai, China) following the manufacturer's instructions. Cells were seeded in 96-well plates at a density of 5 × 10^3^ cells/well. After treatment, CCK8 solution was added to each well and incubated with cells for 3 h. Then, the absorbance value was assessed at 450 nm by the application of a microplate reader (Bio-Rad, Germany). The relative survival rate of the cells was compared with that of the blank control group.

### Analysis of the mitochondrial membrane potential by JC-1 staining

The mitochondrial membrane potential (MMP) was assessed by JC-1 staining (Beyotime, Shanghai, China) following the manufacturer's protocol, and carbonyl cyanide 3-chlorophenylhydrazone (CCCP) at 10 mM was used to treat cells for 20 mins as a positive control. Briefly, 1 mL of JC-1 was added directly to 1 mL of culture medium and incubated for 15 mins at 37 °C. At the end of incubation, the supernatant was discarded, and the cells were then washed three times with JC-1 staining buffer. Finally, the treated cells were observed under a confocal microscope (Thermo Fisher Scientific, WA, USA). The JC-1 monomer-recorded green fluorescence was quantified at 490/530 nm (excitation/emission), whereas the JC-1 complex-recorded red fluorescence was detected at 525/590 nm. (excitation/emission).

### Acridine orange/ethidium bromide (AO/EB) double staining

Cells were seeded and treated with TNF-α and SP for 24 h, as described above. AO/EB was purchased from Sigma Aldrich (Shanghai, China). As previously described 40, the AO/EB mixture was prepared with 100 mg/L AO and 100 mg/L EB in PBS. The cells were centrifuged and suspended in PBS, and 100 μL of cell suspension with 4 μL of AO/EB staining solution was dripped onto glass slides for 10 mins, which were covered with a coverslip and immediately placed under a fluorescence microscope for observation (Nikon Corp., Tokyo, Japan). Digital images were recorded with a Coolpix camera using a Lucia image analyzer (Nikon Italia, Italy).

### mtDNA transfection and depletion

The mtDNA was acquired from Caco-2 cells using a mtDNA Extractor CT Kit (Wako Pure Chemical Industries, Japan) based on the manufacturer's guidelines. The mtDNA was stored in boric acid-EDTA buffer (Solarbio, Beijing, China). Cells were transfected with 2.5 μg/mL mtDNA and cultured for 24 h utilizing Lipofectamine 8000 (Beyotime, Shanghai, China) following the manufacturer's instructions. The mtDNA depletion experiment was performed by treating Caco-2 cells with 1.0 μg/mL ethidium bromide (EtBr; Shanghai, Sigma Aldrich) for 48 h.

### Protein docking

The 3D structures of NK1R (UniProt ID: P25103) and STING (UniProt ID: Q86WV6) were acquired from UniProt (https://www.uniprot.org/). The full-length AlphaFold predicted structures are shown as protein structure files. We set appropriate docking parameters and then analyzed the interaction between NK1R and STING using HDOCK for protein-protein docking. A negative docking score indicates a more likely combination model. The confidence score is higher than 0.7, suggesting greater binding affinity of the combined model.

### RNA extraction and quantitative real-time PCR

The qRT-PCR was carried out as previously described 41. Total mRNA was acquired from colon tissues or Caco-2 cells using RNAiso Plus (Takara Bio, Beijing, China) and was reverse transcribed using the HiFiScript cDNA synthesis kit from Vazyme (Jiangsu, China) following the manufacturer's instructions. cDNA was used as the template, and the mRNA level was assessed using SYBR Green Master Mix (CWBIO, Beijing, China) on the ABI PRISM 7500 Fast Sequence Detection System (Applied Biosystems, Shanghai, China). The test was conducted according to the following procedure: 95 °C for 30 s, 95 °C for 5 s, and 60 °C for 30 s for 40 cycles. To ensure typical amplification curves and single-peaked melting curves, indicating the presence of a single amplification product for each primer pair (gel electrophoresis can be run to check for single amplification if melting curve analysis is not performed), the coefficient of variation within replicate groups should be equal to or less than 10%, and no signal should be detected in the negative control group. GAPDH was used as the internal control gene. Relative mRNA expression levels were calculated with the comparative cycle threshold (Ct; 2-^ΔΔCt^) method.

The quantification of mtDNA in the serum and cell supernatant was conducted by qPCR as previously described 42. Total DNA was isolated using the QIAamp DNA Mini Kit (Qiagen, Valencia, CA). The copy numbers of NADH dehydrogenase subunit 1 (ND1), cytochrome c oxidase subunit 2 (COXII), cytochrome B (CytB), and NADH dehydrogenase subunit 2 (ND2) were normalized using nuclear 18S ribosomal RNA gene copies. The mouse and human sequences of primers are shown in Table S1 and Table S2, respectively.

### Immunoblotting

As previously described in the literature 43, cells and colon tissues were lysed with radioimmunoprecipitation assay (RIPA) lysis buffer (CWBIO, Beijing, China) supplemented with 1% protease and phosphatase inhibitor cocktails (CWBIO, Beijing, China) on ice and centrifuged at 14000 × g for 15 mins at 4 °C. The protein content was detected utilizing a BCA protein assay kit (Beyotime, Shanghai, China) and was analyzed as pg/mg. The proteins were separated through sodium dodecyl sulfate-polyacrylamide gel electrophoresis (SDS-PAGE) and then transferred to a polyvinylidene difluoride membrane (Millipore, USA), followed by the block in TBS containing 0.1% Tween 20 (TBST) supplemented with 5% nonfat milk at room temperature for 2 h. The membranes were incubated at 4 °C with primary antibodies against SP (abs137008, 1:1000, Absin, Shanghai, China), NK1R (DF7438, 1:500, Affinity, Michigan, USA), cGAS (A8335, 1:1000, ABclonal, Wuhan, China), STING (NBP2-24683, 1:1000, NOVUS, Missouri, USA), phospho-interferon regulatory factor 3 (p-IRF3, AF2436, 1:1000, Affinity, Wuhan, China), phospho-TANK-binding kinase 1 (p-TBK1, AF8153, 1:1000, Affinity, Wuhan, China), glutathione peroxidase 4 (GPX4, bs-3884R, 1:1000, Bioss, Beijing, China), cyclooxygenase 2 (COX-2, WL01750, 1:500, Wanleibio, Shenyang, China), and ferritin Heavy Chain 1 (FTH1, WL05360, 1:500, Wanleibio, Shenyang, China). After three washes with TBST, the membrane was incubated with HRP-conjugated goat anti-mouse IgG (H&L) (A0216, 1:1000, Beyotime, Beijing, China) or HRP-conjugated goat anti-rabbit IgG (H&L) (A0208, 1:1000, Beyotime, Beijing, China) at room temperature for 1 h. Mouse anti-β-actin (50201, 1:1000, Kemei Borui, Beijing, China) was used to normalize the protein level. The signals were visualized with an enhanced chemiluminescence (ECL) reagent (Tanon, China). Images were obtained with a gel imaging system (Tanon 4800, China). The intensity of the protein band was analyzed by ImageJ software (Maryland, USA).

### Immunofluorescence (IF) staining

Immunofluorescence staining was performed exactly according to a previous study 44. The colon tissue sections were fixed in 4% paraformaldehyde for 30 mins and then permeabilized with 0.1% Triton X-100 for 20 mins. The sections were blocked with donkey serum for 30 mins at room temperature and then incubated with primary antibodies against microtubule associated protein 2 (MAP-2, NB300-213, 1:100, NOVUS, Missouri, USA), C-FOS (sc-166940, 1:50, Santa Cruz, Dallas, USA), SP (abs137008, 1:100, Absin, Shanghai, China), CD206 (ab64693, 1:1000, Abcam, Cambridge, MA, USA), CD68 (ab125212, 1:1000, Abcam, Cambridge, MA, USA), NK1R (DF7438, 1:200, Affinity, Michigan, USA), STING (NBP2-24683, 1:200, NOVUS, Missouri, USA) and E-cadherin (PB9561, 1:200, Boster, China) overnight at 4 °C. For the cell immunofluorescence staining method as described above, the primary antibodies used were anti-NK1R (DF7438, 1:200, Affinity, Michigan, USA), anti-cGAS (A8335, 1:200, ABclonal, Wuhan, China), and anti-STING (NBP2-24683, 1:200, NOVUS, Missouri, USA) and incubated overnight at 4 °C. After washing with PBS, the cells and sections were incubated with biotinylated secondary antibodies at 4 °C. Then, the sections and cells were incubated with fluorescent antibodies at 4 °C for 24 h and washed twice with PBS. Finally, the cells and sections were treated with 4′,6-diamidino-2-phenylindole (DAPI) (SouthernBiotech, Birmingham, AL, USA) and then observed with a confocal laser-scanning microscope (Nikon, Tokyo, Japan).

### Statistical analysis

All the experimental data are presented as the mean ± standard error (SEM). Statistical significance between groups was assessed by the two-tailed Student's t-test or one-way analysis of variance (ANOVA). At least three independent experiments were conducted to confirm the results. Statistical analysis and graphs generation were performed using GraphPad Prism 8.0 software. Spearman's correlation coefficient (r) was used to calculate the relationship between serum mtDNA levels and cytokine contents. Statistical significance values were defined as follows: ^*^p < 0.05 or ^#^*p* < 0.05.

## Results

### SP-positive neurons are activated, and SP expression is increased in the colon tissue of DSS-induced colitis mice

**Figure 1:**
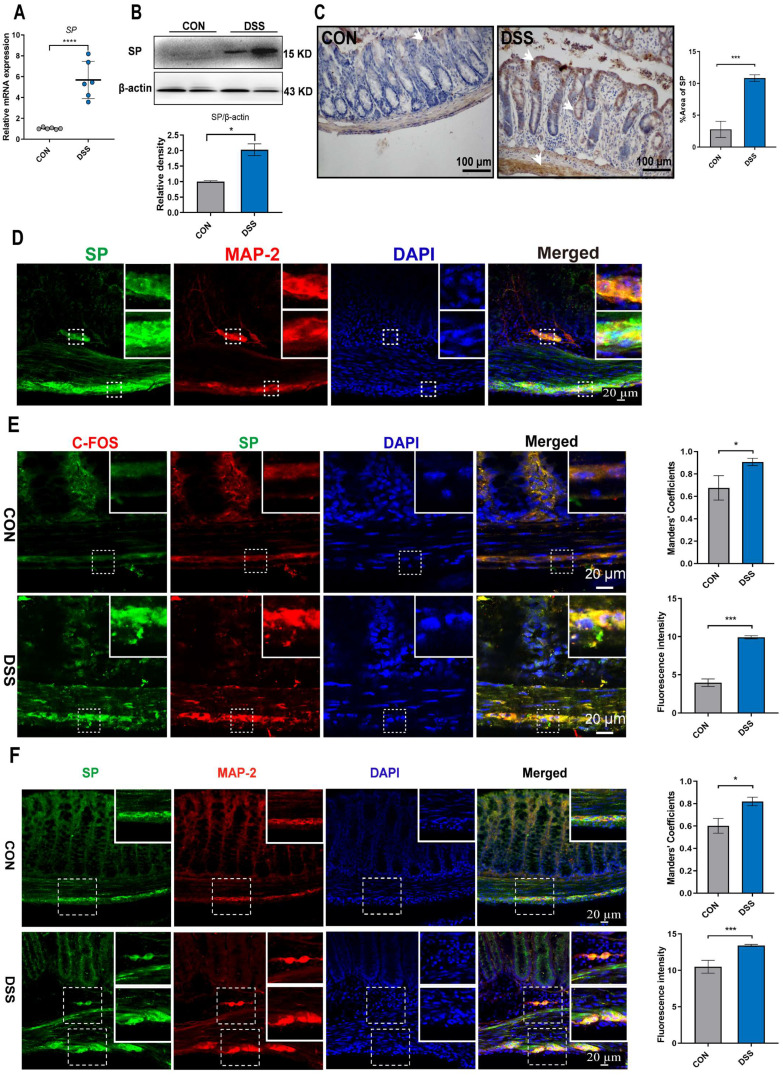
**SP-positive neurons are activated, and SP expression is increased in the colon tissue of colitis mice. (A)** The mRNA expression of *SP* in colon tissue by qRT-PCR. **(B)** Protein expression of SP was measured in colon tissue by western blot and analysis of quantification. **(C)** Representative immunohistochemistry images of SP in colon tissue and statistical analysis. **(D)** SP-positive neurons were detected by double immunofluorescence staining for SP (green)/MAP-2 (red) in colon tissues and statistical analysis. Nuclei were stained with DAPI (blue). Scale bar = 20 μm.** (E)** Double immunofluorescence staining for C-FOS (red) and SP (green) in colon tissues and statistical analysis. Nuclei were stained with DAPI (blue). Scale bar = 20 μm. **(F)** Localization of SP (green) and MAP-2 (red) in colon tissues and statistical analysis, as evaluated by double immunofluorescence staining. Nuclei were stained with DAPI (blue). Scale bar = 20 μm. Data are representative of one experiment repeated three independent times. Data are expressed as the mean ± SEM; n = 6. ^*^p<0.05 and ^****^p<0.0001.

**Figure 2:**
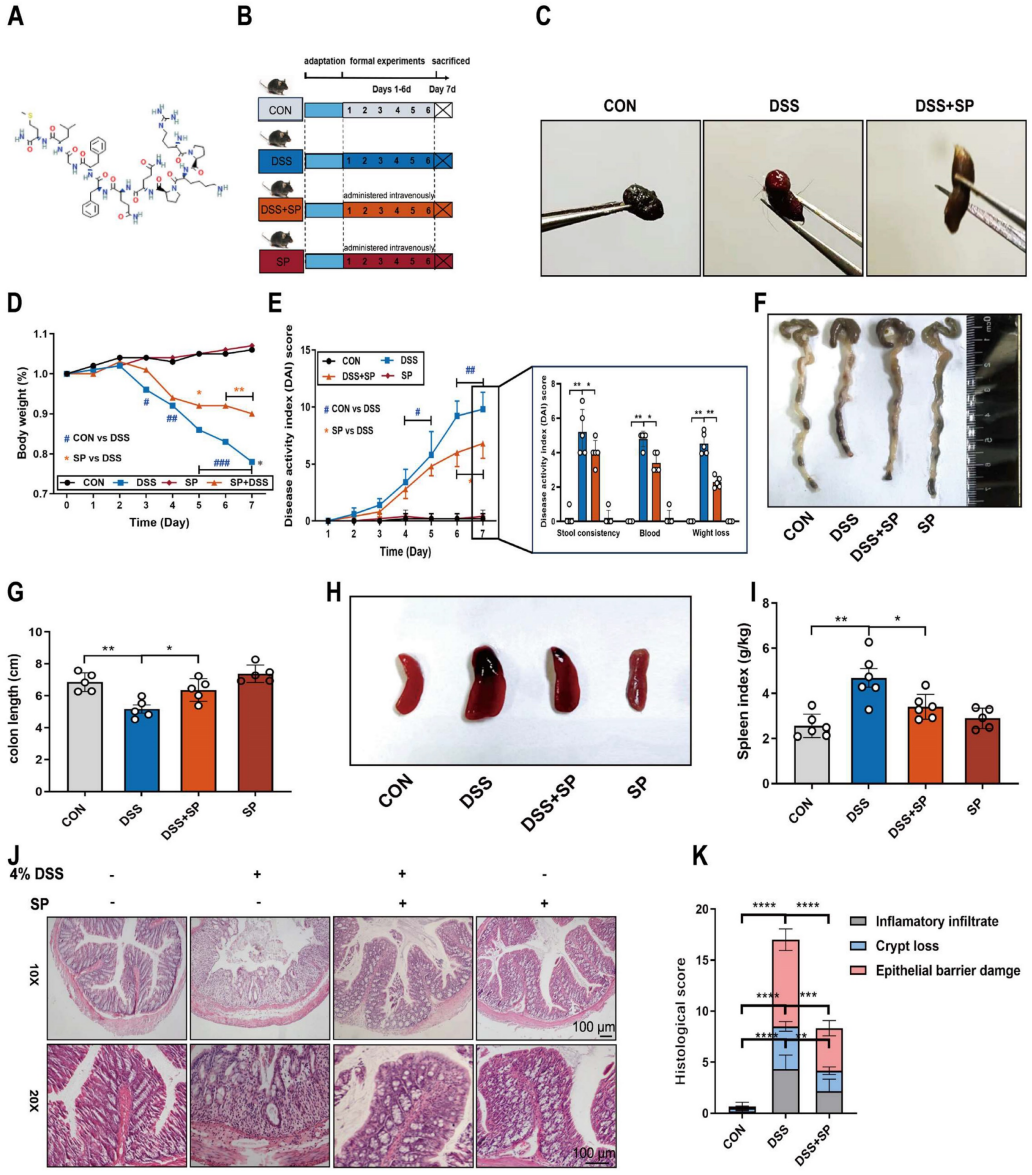
**SP alleviates pathological indexes in DSS-induced colitis mice. (A)** Chemical structure of substance P. **(B)** Experimental design and treatment strategy. **(C)** Fecal phenotype. **(D)** Changes in body weight. **(E)** Disease activity index. **(F)** Photographs of colon length. **(G)** The quantification of colon length. **(H, I)** Images spleen and spleen index. **(J, K)** Representative images of H&E sections and histological score. Scale bar = 100 μm. Data are obtained from three independent experiments, and expressed as the mean ± SEM; n = 5. ^*^p<0.05, ^**^p<0.01 and ^****^p<0.0001.

Mice were subjected to a 6-day treatment of DSS through their drinking water to induce severe colitis, resulting in weight loss, diarrhea, and hematochezia (Figure 2D, E), confirming the successful establishment of the colitis model. To investigate the influence of SP on intestinal barrier function in colitis, we examined the expression of endogenous SP. The results showed elevated the mRNA and protein levels of SP in mice with colitis compared with the CON group (Figure 1A, B). These findings were further supported by immunohistochemistry analysis, which verified an increase in SP expression, particularly within the epithelium and muscular layer of the intestine (Figure 1C).

Given the evidence that SP could be generated from SP-positive neurons in the intestine 9, we conducted double immunofluorescence staining for SP/MAP-2. As shown in Figure 1D, numerous SP-positive neurons were observed in both the myenteric and submucosal plexuses. Notably, SP-positive nerve fibers extended to the lamina propria and intestinal epithelium, suggesting a potential critical role of SP in regulating intestinal mucosal function. Furthermore, immunofluorescence staining for SP/C-FOS revealed robust activation of SP-positive neurons after DSS exposure (Figure 1E). We further found that mice treated with DSS exhibited a remarkable increase in the number of SP-positive neurons in the submucosal and myenteric plexuses compared to CON mice (Figure 1F). These data support our hypothesis that SP may have protective effects on DSS-induced colitis.

### SP alleviates pathological indexes and intestinal barrier dysfunction in DSS-induced colitis mice

To investigate the potential efficacy of SP in colitis, mice were treated with 4% DSS to cause acute colitis, with or without SP treatment. The experimental procedure is outlined in Figure 2B. Remarkably, administration of exogenous SP mitigated DSS-induced clinical performance (Figure S1). Consistently, SP treatment led to significant improvements in fecal phenotype and body weight loss (Figure 2C, D). Meanwhile, SP administration resulted in reduced DAI scores in DSS-challenged mice, as evidenced by relieved weight loss, gross bleeding, and stool consistency (Figure 2E). Additionally, the colon shortening (Figure 2F, G) and splenomegaly (Figure 2H, I) were recovered in mice simultaneously treated with DSS and SP, compared with the DSS group. Histological examination revealed that DSS-induced colon pathological alterations were substantially improved following SP treatment, as indicated by lower histological scores (Figure 2J, K). Notably, no apparent pathological changes were observed between the SP treatment alone and the CON group.

**Figure 3:**
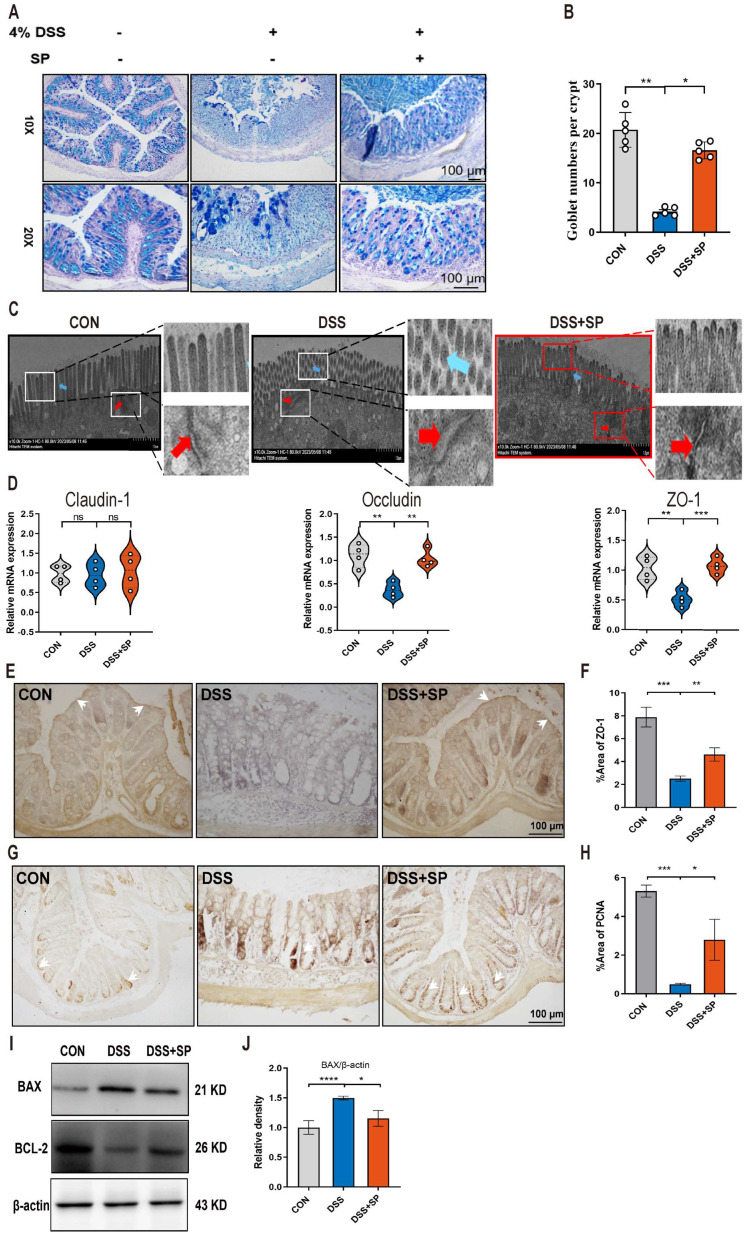
**SP alleviates intestinal barrier dysfunction in DSS-induced colitis mice. (A, B)** AB-PAS staining of colon tissue and quantification of goblet cell number. Scale bar=100 μm. **(C)** TJ proteins and microvilli length of colonic ultrastructure by TEM. Microvilli as indicated by light blue arrows; TJ protein as indicated by red arrows. Scale bar = 1 μm. **(D)** The mRNA levels of *Claudin-1*, *Occludin*, and *ZO-1* were tested in colon tissue by qRT-PCR. **(E, F)** Representative immunohistochemistry images of ZO-1 in colon tissue and statistical analysis. Scale bar = 100 μm.** (G, H)** Immunohistochemistry image of PCNA in colon tissue and statistical analysis. Scale bar = 100 μm.** (I, J)** The expression of BAX and Bcl-2 in colonic tissue, as determined by western blotting and quantitative density data. Data are obtained from three independent experiments, and expressed as the mean ± SEM; n = 4 or 5. ^*^p<0.05, ^**^p<0.01 and ^****^p<0.0001.

The intestinal barrier is crucial for preserving tissue integrity and promoting gut health. Impairment of intestinal integrity and protein dysregulation have been identified as primary factors contributing to the development of UC and colitis models 45. Our data demonstrated that mice treated with SP exhibited higher numbers of goblet cells and increased mucus expression, as observed through AB-PAS staining, compared to the DSS-induced mice (Figure 3A, B). Tight junction (TJ) proteins function as mechanical barriers, preventing the entry of external pathogens 46. As shown in Figure 3C, TEM analysis revealed irregular colonic epithelium with intercellular spaces, shortened microvilli, and disrupted TJ proteins in the DSS group. However, SP effectively counteracted these alterations. RT-qPCR analysis showed that the mRNA levels of TJ proteins, such as *ZO-1* and *Occludin*, were elevated in the SP group compared to the DSS group, whereas *Claudin-1* expression showed no significant difference (Figure 3D). Immunohistochemistry results showed a similar trend in the expression of ZO-1 (Figure 3E, F). The balance between intestinal epithelial cell proliferation and apoptosis is crucial for maintaining intestinal health 47. We next found that SP treatment increased the protein levels of PCNA (Figure 3G, H) and B-cell lymphoma-2 (BCL-2) while reducing the level of Bcl-2-associated x (BAX) (Figure 3I, J). The above results suggest that SP effectively preserved the integrity of the epithelial barrier, regulated cell proliferation and apoptosis, and maintained intestinal homeostasis.

### SP inhibits the infiltration of macrophages and regulates inflammatory cytokine expression *in vivo* and *in vitro*

**Figure 4:**
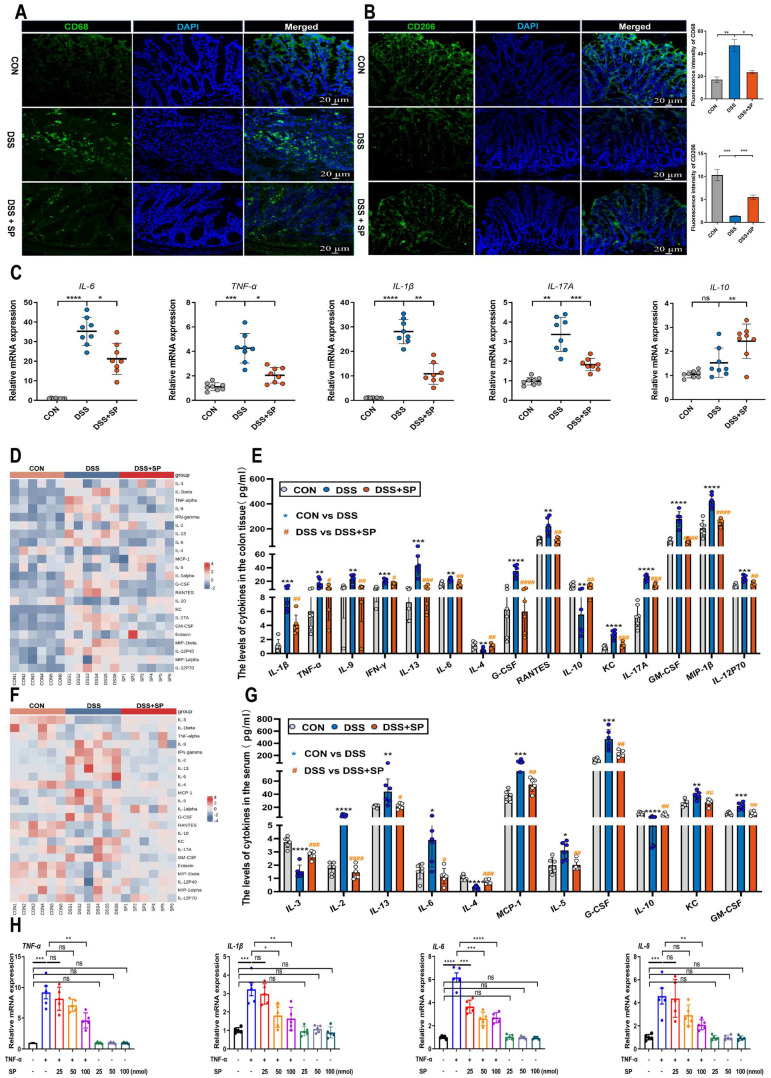
**SP inhibits the infiltration of macrophages, promotes M2 macrophage polarization, and regulates inflammatory cytokine expression. (A, B)** Immunofluorescence analysis for CD68 and CD206 in the colon tissue and statistical analysis. Nuclei were stained with DAPI (blue). Scale bars = 20 µm. **(C)** The mRNA levels of inflammatory cytokines as indicated by *IL-6*, *TNF-α*, *IL-17A*, *IL-1β* and *IL-10* in the colon tissue. **(D, E)** Cytokines protein expression in the colon tissue of mice by Luminex technology and level analysis. **(F, G)** Serum cytokines levels in each group were determined by Luminex technology and data analysis. **(H)** RT-qPCR assay of inflammatory cytokines (*TNF-α*, *IL-1β*, *IL-6* and *IL-8*) in TNF-α-stimulated Caco-2 cells treated with/without SP (25, 50, and 100 nmol) for 24 h. Data are obtained from three independent experiments, and expressed as the mean ± SEM; n = 5-8. ^*^p<0.05, ^**^p<0.01, ^***^p<0.001 and ^****^p<0.0001.

When the intestinal mucosa is injured, a cascade of immune responses is triggered, with macrophages playing a crucial regulatory role in the inflammatory response of patients with IBD 48. Immunofluorescence analysis revealed a significant increase in the expression of CD68 (a macrophage marker) and a decrease in the expression of CD206 (an M2 macrophage marker) in colon tissue after DSS treatment. However, the administration of SP reversed these changes (Figure 4A, B). These results demonstrate that SP can reduce the infiltration of macrophages and promote M2 macrophage polarization.

Prolonged or excessive proinflammatory factors can disrupt the function of the intestinal epithelial barrier and further exacerbate intestinal damage 49. To validate the anti-inflammatory effect of SP, we assessed the expression of inflammatory factors using RT-qPCR. The results demonstrated that SP decreased mRNA levels of *IL-6*, *TNF-α*, *IL-17A*, and *IL-1β*, but enhanced the mRNA level of *IL-10* in mice with DSS-induced colitis (Figure 4C). There was no significant difference in the mRNA expression of inflammatory cytokines between the CON group and the SP alone group (Figure S2). We subsequently examined several cytokines and chemokines in colon tissue and serum by Luminex multiplex assays. Following the challenge with DSS, the contents of IL-1β, TNF-α, IL-9, IFN-γ, IL-13, IL-6, G-CSF, RANTES, KC, IL-17A, GM-CSF, MIP-1β, and IL-12P70 were elevated in colon tissue, while the levels of IL-4 and IL-10 were sharply reduced. However, SP treatment effectively reversed these changes (Figure 4D, E). Additionally, in the serum, DSS-treated mice exhibited increased levels of IL-2, IL-13, IL-6, MCP-1, IL-5, G-CSF, KC, and GM-CSF in DSS-treated mice, along with decreased levels of IL-3, IL-4 and IL-10. Notably, SP remarkably mitigated these alterations in inflammatory cytokines (Figure 4F, G). These findings suggest that SP can reduce inflammation by modulating the balance between proinflammatory cytokines and anti-inflammatory factors.

To further investigate the effects of SP, we cultured Caco-2 cells and exposed them to different doses of TNF-α for 24 h to mimic an inflammatory cellular state. Upon stimulation with TNF-α at a concentration of 100 ng/mL, the mRNA levels of *IL-6* and *TNF-α* were significantly enhanced (Figure S3A), suggesting the successful establishment of a cell inflammation model. Following that, the cytotoxicity of SP was assessed using a CCK-8 assay. As depicted in Figure S3B, SP at concentrations equal to or below 100 nmol for 24 h did not affect cell survival, demonstrating no significant toxicity to Caco-2 cells. AO/EB staining illustrated that SP significantly increased the number of live cells while reducing the number of apoptotic cells compared to TNF-α exposure alone (Figure S3C). Subsequently, RT-qPCR results showed that SP dose-dependently declined the mRNA expressions of proinflammatory cytokines, including *TNF-α*, *IL-1β*, *IL-6*, and *interleukin 8* (*IL-8*), in response to TNF-α exposure. Intriguingly, the inhibitory effect of SP was most pronounced at a concentration of 100 nmol (Figure 4H), which indicates the capacity of SP to suppress cytokine storms induced by TNF-α *in vitro*.

### SP down-regulates DSS or TNF-α-induced cGAS-STING signaling pathway activation *in vivo and in vitro*

**Figure 5:**
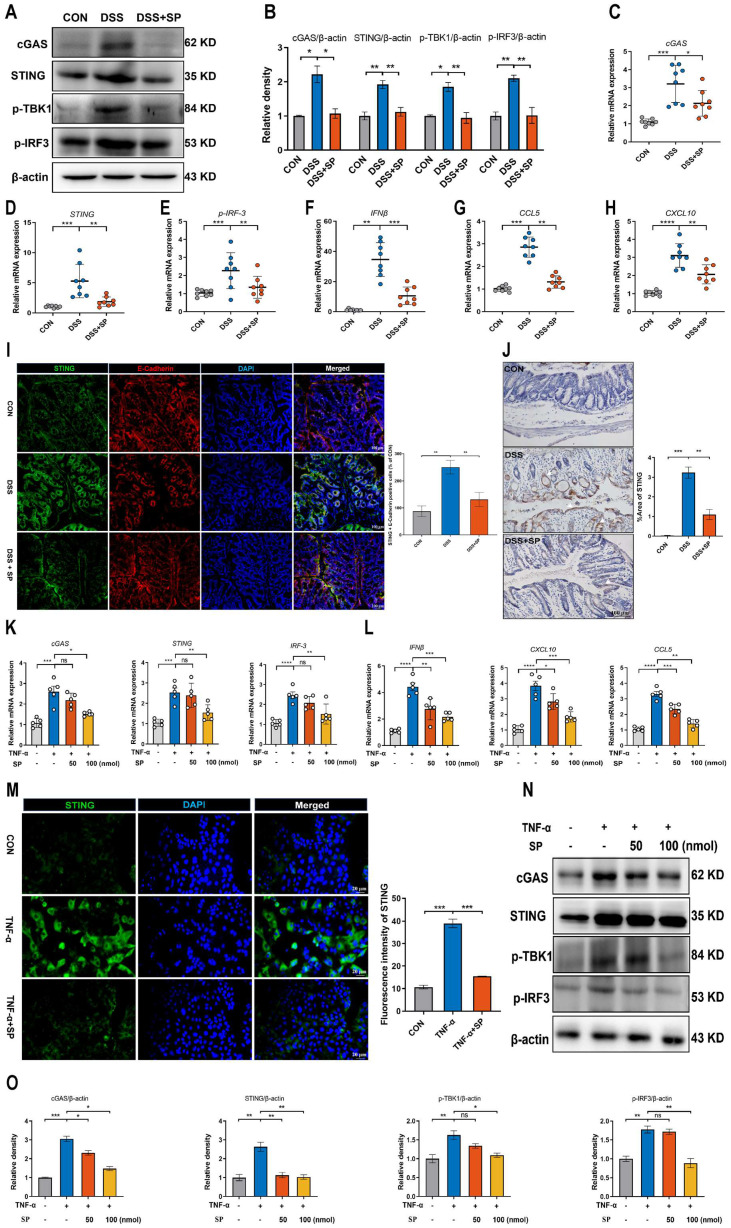
** SP down-regulates DSS or TNF-α-induced cGAS-STING signaling pathway *in vivo and in vitro.* (A)** The expressions of proteins related to the cGAS-STING signal pathway were examined by western blot in the colon tissue. **(B)** Protein expression quantification. **(C-E)** Relative mRNA levels of *cGAS*, *STING*, and *p-IRF3* in the colon tissue. **(F-H)** The mRNA expressions of downstream cytokines (*IFNβ*, *CXCL10*, and *CCL5*) in the colon tissue. **(I)** Double immunofluorescence staining for STING (green)/E-cadherin (red) in colon tissues and statistical analysis. Scale bar = 100 μm. **(J)** Immunohistochemistry images of STING in colon tissue and statistical analysis. Scale bar = 100 μm. **(K)** Relative mRNA levels of cGAS-STING related molecules (*cGAS*, *STING*, and *p-IRF3*) and **(L)** downstream cytokines (*IFNβ*, *CXCL10*, and *CCL5*) in Caco-2 cells. **(M)** Pictures of immunofluorescence staining for STING (green) and DAPI nuclear staining (blue) in Caco-2 cells and statistical analysis. Scale bars = 20 µm.** (N)** Relative protein levels of cGAS, STING, p-TBK1, and p-IRF3 in Caco-2 cells, as identified by western blotting. **(O)** Representative blots were quantitated by ImageJ software. Data are obtained from three independent experiments, and expressed as the mean ± SEM; n = 5 or 8. ^*^p<0.05, ^**^p<0.01, ^***^p<0.001, and ^****^p<0.0001.

It has been demonstrated that the cGAS-STING signaling pathway plays a vital role in triggering innate immune responses and regulating gut homeostasis as well as the development of intestinal disorders 28. We supposed that SP could regulate the cGAS-STING pathway to relieve the inflammatory response. The results confirmed that the protein levels of cGAS, STING, p-TBK-1, and p-IRF3 were significantly augmented in the DSS group, but their expression was effectively suppressed by SP administration (Figure 5A, B). Consistent with the protein expression, SP reversed the DSS-induced upregulation of mRNA levels of *cGAS*, *STING*, and *p-IRF3* (Figure 5C-E), as well as downstream cytokines such as *interferon-beta* (*IFNβ*), *c-x-c motif chemokine ligand 10* (*CXCL10*), and *chemokine (c-c motif) ligand 5* (*CCL5*) (Figure 5F-H). Immunofluorescence double-staining for STING and E-cadherin was performed on colon tissues. The results revealed that SP significantly suppressed the STING-positive expression induced by DSS in intestinal epithelial cells (Figure 5I). In addition, immunohistochemistry assays verified that SP dramatically diminished the DSS-induced increase in STING expression (Figure 5J).

Likewise, *in vitro* studies consistently confirmed that SP treatment could dampen the cGAS-STING signaling pathway. Compared to the TNF-α exposure alone, SP at concentrations of 50 and 100 nmol significantly reduced the mRNA levels of *cGAS*, *STING*, and *p-IRF3* in a dose-dependent manner (Figure 5K). Furthermore, SP treatment dose-dependently reduced the mRNA levels of *IFNβ*, *CXCL10*, and *CCL5* (Figure 5L). Immunofluorescence results revealed that the activation of STING induced by TNF-α was strongly suppressed by SP, weakening the intensity of STING-positive signaling (Figure 5M).

Consistently, SP effectively decreased the protein expression of cGAS, STING, p-TBK1, and p-IRF3 in a dose-dependent manner (Figure 5N, O). Based on these findings, SP at a concentration of 100 nmol for 24 h was employed in subsequent tests. These results provide valuable insights into the mechanisms underlying the protective effects of SP against colitis by suppressing the cGAS-STING pathway.

### SP relieves DSS or TNF-α-induced mitochondrial damage and prevents mtDNA release *in vivo and in vitro*

**Figure 6:**
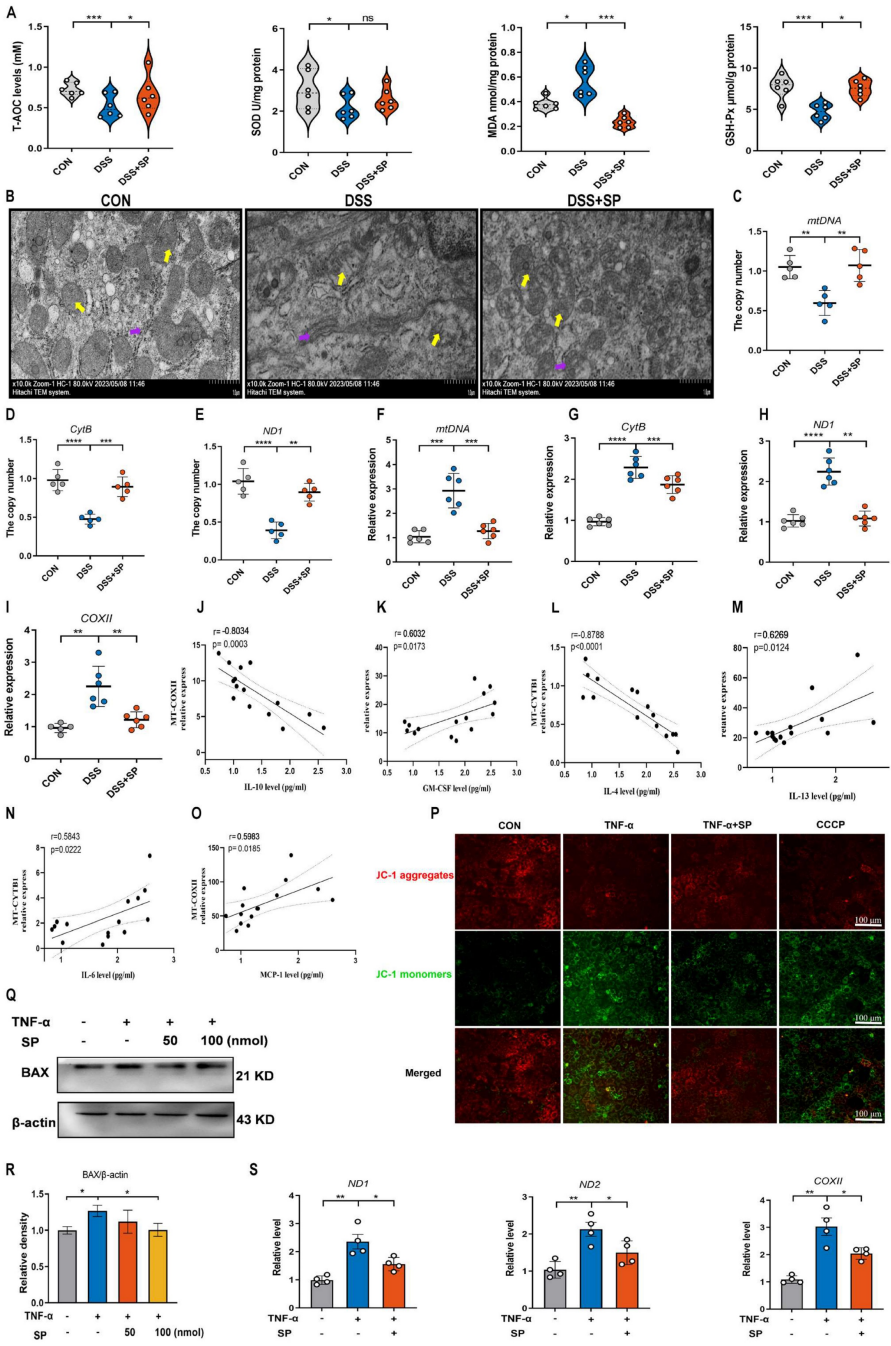
**SP relieves DSS or TNF-α-induced mitochondrial damage and prevents mtDNA release. (A)** The levels of T-AOC, MDA, GSH-Px, and SOD in the colon tissue. **(B)** The mitochondrial structure of the colon tissue was observed by TEM. Mitochondria as indicated by yellow arrows; Rough endoplasmic reticulum as indicated by purple arrows. Scale bars = 1 µm. **(C-E)** Relative mRNA levels of mitochondrial coding genes (*mtDNA*, *CytB*, and *ND1*) in colon tissue of different groups. **(F-I)** Relative expression of mtDNA-related genes (*mtDNA*, *CytB*, *ND1*, and *COXII*) in the serum. **(J-O)** Spearman analysis of the correlation between serum mtDNA level and cytokine content. **(P)** Representative picture of JC-1 staining in TNF-α-treated Caco-2 cells incubated with SP (100 nmol) for 24 h. **(Q, R)** Western blot analysis of BAX protein level in Caco-2 cells and relative expression levels. **(S)** The mRNA expressions of *ND1*, *ND2*, and *COXII* in the culture supernatant of TNF-α-stimulated Caco-2 cells treated with/without SP. Data are obtained from three independent experiments, and expressed as the mean ± SEM; n = 4-6. ^*^p<0.05, ^**^p<0.01, ^***^p<0.001 and ^****^p<0.0001

A recent study has provided evidence that mtDNA, known as DAMPs, is a primary activator of the cGAS-STING pathway when it is released into the cytosol due to mitochondrial stress and damage 50. As shown in Figure 6A, the DSS group displayed decreased activity of GSH-Px and T-AOC, along with an elevated concentration of MDA compared to the CON group. However, the administration of SP effectively reversed these alterations. Notably, the administration of SP had no significant effect on SOD activity. Since aberrant mitochondrion has been widely observed in the pathological conditions of UC, we also examined the mitochondrial structure of colon tissue by TEM (Figure 6B). DSS-exposed mice exhibited severe mitochondrial impairment characterized by swollen mitochondria, disrupted cristae, and loss of double membrane structure. Conversely, mice treated with SP exhibited a significantly lower incidence of aberrant mitochondria. Additionally, the relative expressions of the mitochondrial-related genes, such as *mtDNA*, *CytB*, and *ND1*, were reduced in DSS-exposed mice, whereas SP remarkably reversed these changes (Figure 6C-E), indicating that SP restored DSS-induced mitochondrial dysfunction. The mtDNA is unstable and easily released from damaged mitochondria into the cytosol under the influence of oxidative stress 26. To estimate whether mtDNA was released into the cytosol, we tested and found that DSS treatment increased the circulating levels of *mtDNA*, *CytB*, *ND1*, and *COXII* in the serum. Conversely, SP effectively suppressed the relative levels of mtDNA-related genes (Figure 6F-I). Moreover, Spearman analysis displayed a significant correlation between cytokine concentrations and mtDNA levels, with a correlation coefficient (|r| > 0.5, *p* < 0.05) (Figure 6J-O), suggesting that mtDNA contributed to the aggravation of colonic inflammation. Subsequently, an *in vitro* study assessed mitochondrial function by MMP assay, with CCCP serving as a positive control. TNF-α exposure significantly disrupted the MMP, as indicated by enhanced green fluorescence and reduced red fluorescence, whereas the administration of SP decreased the number of JC-1-labeled monomers (Figure 6P), suggesting that SP mitigated the loss of MMP and improved mitochondrial function. The dissipated MMP was accompanied by increased expression of BAX, a component of the permeability transition pore that allows mtDNA to leak from the mitochondria 51. *In vitro*, we found that the administration of SP significantly reduced the protein expression of BAX in Caco-2 cells compared to TNF-α stimulation (Figure 6Q, R). Subsequently, the release of mtDNA into the culture supernatant was measured by RT-qPCR. The results revealed that SP notably decreased TNF-α-induced expression of mtDNA-encoded genes, such as *ND1*, *ND2*, and *COXII*, which was consistent with the *in vivo* results (Figure 6S). These findings imply that SP represses the accumulation of mtDNA in the cytosol.

### SP suppresses the mtDNA-cGAS-STING signaling pathway in TNF-α-treated Caco-2 cells

**Figure 7:**
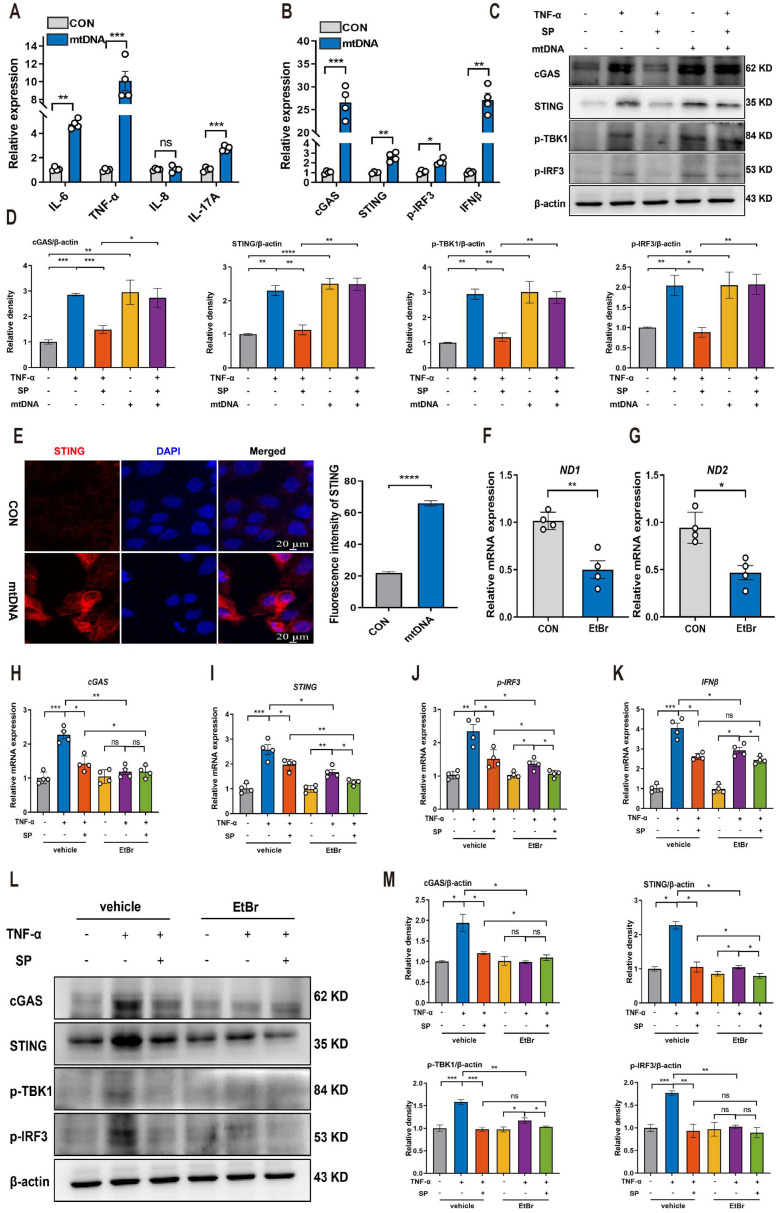
**SP suppresses the mtDNA-cGAS-STING signaling pathway in TNF-α-treated Caco-2 cells. (A)** The mRNA levels of *IL-6*, *TNF-α*, *IL-8*, and *IL-17A* in Caco-2 cells transfected with mtDNA (2.5 μg/mL) for 24 h. **(B)** Relative mRNA levels of *cGAS*, *STING*, *p-IRF3*, and *IFNβ* in Caco-2 cells transfected with mtDNA. **(C)** Western blot analysis of cGAS, STING, p-TBK1, and p-IRF3 protein expressions in Caco-2 cells transfected with mtDNA. **(D)** Statistical analysis of the bands normalized to β-actin. **(E)** Immunofluorescence staining for STING (red) and DAPI nuclear staining (blue) in Caco-2 cells transfected with mtDNA and statistical analysis. Scale bars = 20 µm. **(F, G)** The relative expressions of ND1 and ND2 in Caco-2 cells treated with EtBr (1 μg/mL) for 48 h. **(H-K)** The mRNA expressions of *cGAS*, *STING*, *p-IRF3*, and *IFNβ* in TNF-α-stimulated Caco-2 cells incubated with EtBr with/without SP. **(L, M)** The protein levels of cGAS, STING, p-TBK1, and p-IRF3 in TNF-α-stimulated Caco-2 cells incubated with EtBr with/without SP and quantitative density data. Data are obtained from three independent experiments, and expressed as the mean ± SEM; n = 4. ^*^p<0.05, ^**^p<0.01, and ^***^p<0.001.

Based on the aforementioned results indicating that SP could prevent mtDNA leakage into the cytoplasm, we proceeded to investigate whether cytosolic mtDNA induced by TNF-α could activate innate immunity, thus elucidating the potential mechanism by which SP regulated the cGAS-STING pathway. Caco-2 cells were transfected with extracted mtDNA (2.5 μg/mL) for 24 h using lipofectamine 8000. The results displayed that mtDNA significantly upregulated the mRNA expressions of *IL-6*, *TNF-α*, and *IL-17A* (Figure 7A).

Furthermore, the mRNA levels of *cGAS*, *STING*, *p-IRF3*, and *IFNβ* were markedly increased following mtDNA treatment (Figure 7B). Western blot analysis demonstrated that mtDNA transfection effectively activated cGAS, resulting in increased protein levels of STING, p-TBK1, and p-IRF3. Intriguingly, mtDNA treatment markedly decreased or even abolished the inhibitory influence of SP on the cGAS-STING pathway (Figure 7C, D). Immunofluorescence staining also confirmed the activation of the STING-positive signal in Caco-2 cells transfected with mtDNA (Figure 7E).

To provide further compelling evidence that SP inhibits the cGAS-STING pathway by suppressing the release of mtDNA, we employed EtBr at a concentration of 1.0 μg/mL for 48 h to block mtDNA replication, which contributed to a reduced copy number of *ND1* and *ND2*, confirming the successful depletion of mtDNA (Figure 7F, G). Notably, the mRNA levels of *cGAS*, *STING*, *p-IRF3* and *IFNβ* were dramatically suppressed upon EtBr treatment in TNF-α-induced Caco-2 cells (Figure 7H-K).

Consistently, the western blot results verified that the depletion of mtDNA significantly decreased the protein expressions of cGAS, STING, p-TBK1, and p-IRF3 induced by TNF-α (Figure 7L, M). As a result, it is concluded that SP downregulates the cGAS-STING pathway by preventing the release of mtDNA into the cytosol. Interestingly, even in the absence of mtDNA, SP still could repress the mRNA expressions of *STING*, *p-IRF3*, and *IFNβ*, as well as protein levels of STING and p-TBK1, which suggests a potential regulatory effect of SP on the STING pathway through other signaling cascades.

### SP could directly repress the activation of the STING pathway upon DMXAA and SR717 treatment

**Figure 8:**
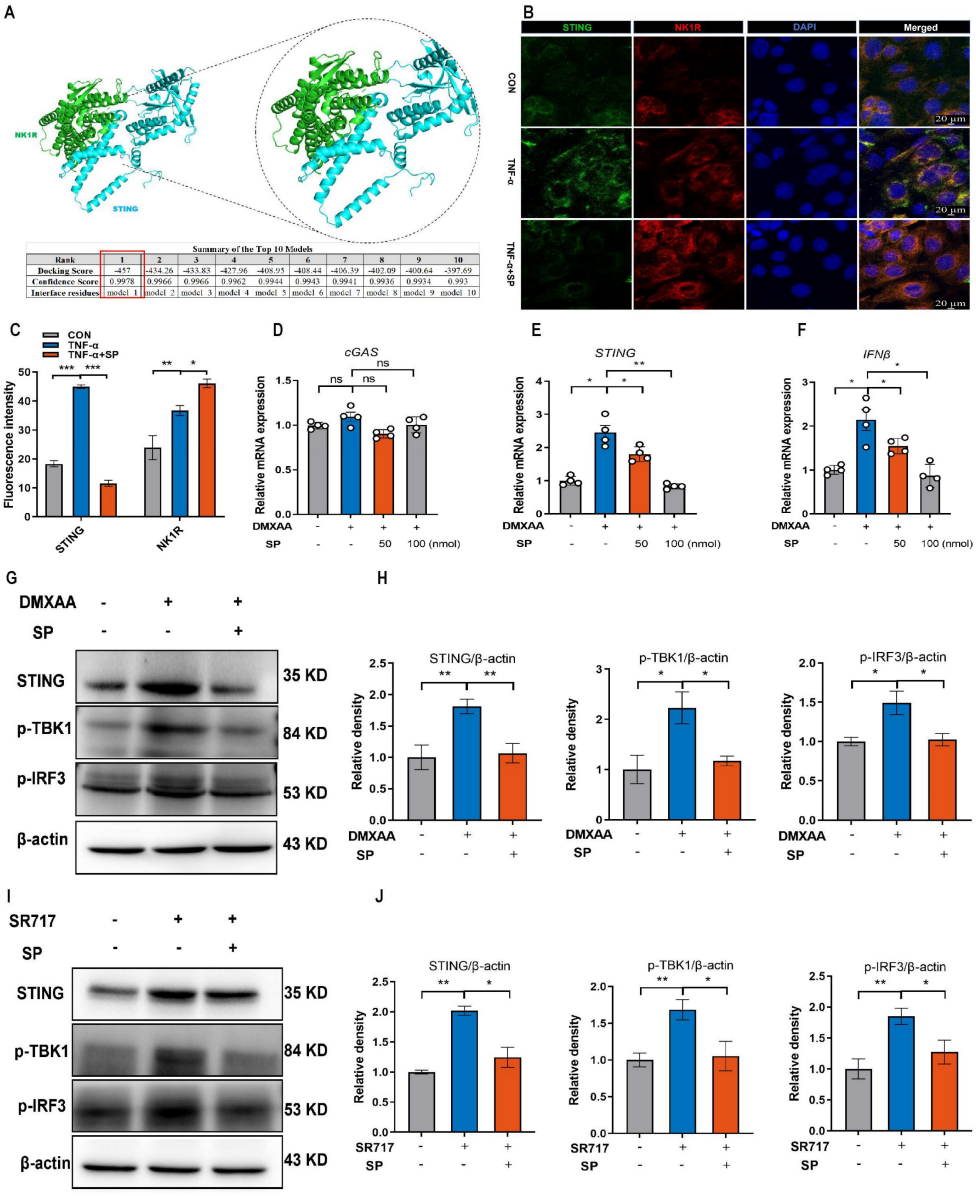
**SP could directly repress the activation of the STING pathway upon DMXAA and SR717 treatment. (A)** The protein docking for NK1R and STING. **(B, C)** Immunofluorescence double-staining for STING (green) and NK1R (red) as well as statistical analysis. DAPI nuclear staining (blue). Scale bars = 20 µm.** (D-F)** The mRNA levels of *cGAS*, *STING*, and *IFNβ* in Caco-2 cells treated with SP and DMXAA (80 μg/mL) for 24 h. **(G, H)** Relative protein levels of STING, p-TBK1, and p-IRF3 in Caco-2 cells treated with DMXAA and SP. **(I, J)** The protein expressions of STING, p-TBK1, and p-IRF3 in Caco-2 cells treated with SP and SR717 (4 μM) for 24 h and their quantitative density data. Data are obtained from three independent experiments, and expressed as the mean ± SEM; n = 4. ^*^p<0.05 and ^**^p<0.01.

We further explored the mechanism by which SP regulates the STING pathway. Interestingly, the protein docking results showed a strong protein binding affinity between NK1R and the STING protein, with docking scores of -457 and a confidence score of 0.9978 (Figure 8A). Immunofluorescence assay confirmed the colocalization of NK1R and STING in Caco-2 cells (Figure 8B, C), suggesting that SP might directly modulate the STING pathway. To test this hypothesis, Caco-2 cells were treated with different doses of DMXAA (20, 40 and 80 μg/mL). Remarkably, the mRNA levels of *STING* and *IFNβ* dramatically increased following DMXAA treatment at 80 μg/mL, but no significant difference was observed in the mRNA expression of* cGAS* between the control and DMXAA-treated groups (Figure S4). Importantly, SP effectively counteracted the DMXAA-induced elevation in mRNA levels of *STING* and *IFNβ* in a dose-dependent manner (Figure 8D-F). Following that, we also found that SP substantially reversed the DMXAA-induced increases in protein expressions of STING, p-TBK1, and p-IRF3 (Figure 8G, H). To provide further support for these findings, we employed another STING agonist (SR717) at concentrations of 1, 2, 4, 8, and 10 μM for 24 h in Caco-2 cells. Treatment with SR717 at 4 μM activated the STING pathway (Figure S5). However, SP dramatically repressed the SR-717-induced activation of the STING pathway, reducing the protein levels of STING, p-TBK1, and p-IRF3 (Figure 8I, J). The data above provide conclusive evidence that SP directly downregulates the STING pathway.

### SP suppresses DSS or TNF-α-induced ferroptosis of intestinal epithelial cells by inhibiting the cGAS-STING pathway

**Figure 9:**
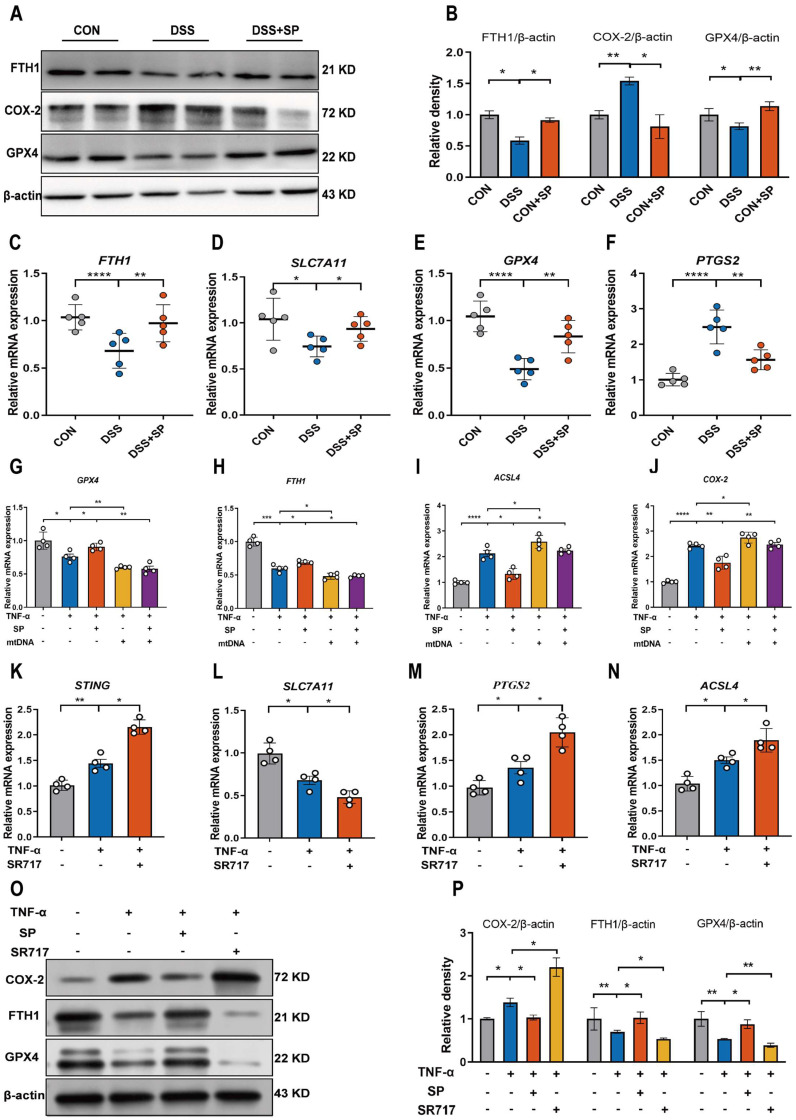
** SP suppresses DSS or TNF-α-induced ferroptosis of intestinal epithelial cells by inhibiting the cGAS-STING pathway. (A, B)** Western blot detected activation of ferroptosis signaling in the colon tissue, as indicated by FTH1, COX-2, and GPX4 normalized to β-actin. **(C-F)** Relative mRNA expressions of *FTH1*, *SLC7A11*, *GPX4* and *PTGS2* in the colon tissue. **(G-J)** The mRNA expressions of *GPX4*, *FTH1*, *ACSL4* and *COX-2* in TNF-α-stimulated Caco-2 cells transfected with mtDNA (2.5 μg/mL) with/without SP for 24 h. **(K-N)** The mRNA levels of *STING*, *SLC7A11*, *PTGS2* and *ACSL4* in TNF-α-treated Caco-2 cells with/without SR717 (4 μM) for 24 h. **(O, P)** Relative protein expressions of ferroptosis markers (COX-2, FTH1 and GPX4) in TNF-α-treated Caco-2 cells with/without SR717. Data are obtained from three independent experiments, and expressed as the mean ± SEM; n = 4 or 5. ^*^p<0.05, ^**^p<0.01 and ^****^p<0.0001.

The cGAS-STING pathway is closely associated with the regulated cell death process known as ferroptosis 52. We hypothesized that SP could dampen the STING pathway and subsequently regulate the ferroptosis process. To verify this possibility, we measured ferroptosis-related markers. Notably, SP significantly elevated the protein levels of GPX4 and FTH1 but decreased COX-2 protein expression compared to the DSS treatment alone (Figure 9A, B). Additionally, RT-qPCR results demonstrated that DSS treatment markedly increased the mRNA expression of *prostaglandin-endoperoxide synthase 2* (*PTGS2*) while reducing the mRNA levels of *FTH1*, *GPX4*, and *solute carrier family 7 member 11* (*SLC7A11*). However, the administration of SP effectively reversed these gene expression changes induced by DSS (Figure 9C-F).

However, it remains unclear whether the inhibitory effects of SP on ferroptosis are dependent on the cGAS-STING pathway. To address this possibility, we transfected Caco-2 cells with mtDNA (2.5 μg/mL) for 24 h to activate the cGAS signal. The results demonstrated that mtDNA transfection accelerated the process of ferroptosis, leading to decreased mRNA levels of* GPX4* and *FTH1*, but elevated levels of *acyl-CoA synthetase long-chain family member 4* (*ACSL4*) and *COX-2*. Importantly, mtDNA transfection reversed the effects of SP on reducing ferroptosis (Figure 9G-J). Furthermore, Caco-2 cells were stimulated with SR717 at 4 μM for 24 h under TNF-α induction. The results displayed that SR717 treatment obviously activated the STING pathway, and promoted ferroptosis, as evidenced by elevated mRNA levels of* PTGS2* and *ACSL4* and reduced mRNA levels of* SLC7A11* in TNF-α-treated Caco-2 cells (Figure 9K-N). Additionally, the western blot assay revealed that SR717 dramatically exacerbated TNF-α-induced ferroptosis, as evidenced by upregulated COX-2 protein expression but downregulated FTH1 and GPX4 protein levels (Figure 9O, P). The above data emphasize that SP could repress cGAS-STING-mediated ferroptosis during colitis.

### SP represses the cGAS-STING pathway to attenuate inflammation and ferroptosis in an NK1R-dependent manner

**Figure 10:**
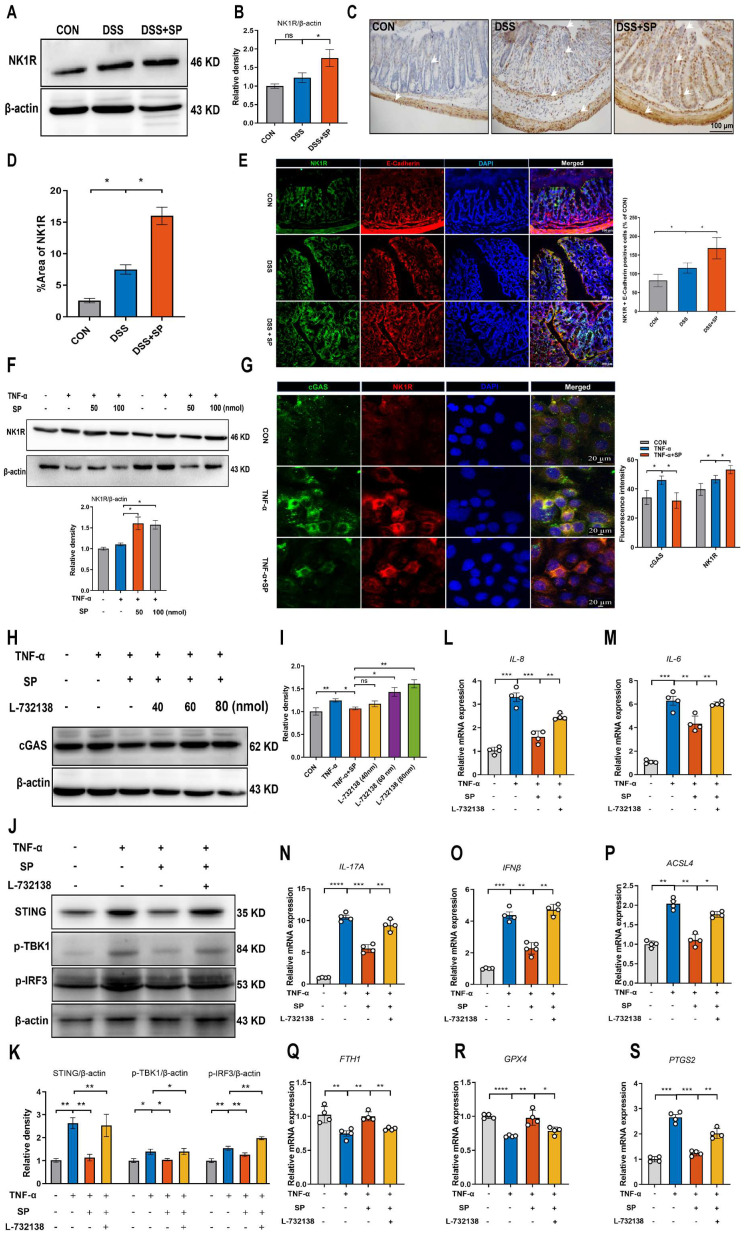
**SP represses the cGAS-STING pathway to attenuate inflammation and ferroptosis in an NK1R-dependent manner. (A, B)** Immunoblotting of NK1R protein in colon tissues and quantitative density data. **(C, D)** NK1R-positive expression in colon tissues was identified using immunohistochemistry staining and statistical analysis. **(E)** Double immunofluorescence staining for NK1R (green)/E-cadherin (red) in colon tissues and statistical analysis. **(F)** The protein expression of NK1R in TNF-α-stimulated Caco-2 cells treated with/without SP (50 and 100 nmol) for 24 h and quantification of relative expression. **(G)** Immunofluorescence double-staining for cGAS (green) and NK1R (red) in TNF-α-stimulated Caco-2 cells. DAPI nuclear staining (blue). Scale bars=20 µm. **(H, I)** The protein level of cGAS was detected when cells were treated with SP (100 nmol) and L-732138 (40, 60 and 80 nmol), as well as quantitative density data. **(J, K)** Western blot analysis of protein expressions (STING, p-TBK1, and p-IRF3) when TNF-α-stimulated cells were treated with/without SP and L-732138 (80 nmol), and quantitative density data. **(L-O)** Relative mRNA expressions of *IL-8*, *IL-6*, *IL-17A* and *IFNβ* in TNF-α-stimulated Caco-2 cells incubated with/without L-732138 and SP, as assessed by RT-qPCR. **(P-S)** The mRNA levels of *ACSL4*, *FTH1*, *GPX4* and *PTGS2* in TNF-α-stimulated Caco-2 cells incubated with/without L-732138 and SP. Data are obtained from three independent experiments, and expressed as the mean ± SEM; n = 4. ^**^p<0.01 and ^***^p<0.001.

Considering the potential modulation of gut inflammation by SP through its receptor NK1R, it is essential to examine NK1R expression during the process of SP alleviating intestinal impairment in colitis. As depicted in Figure 10A, B, mice in the SP group exhibited higher NK1R expression than those in the DSS group. Additionally, immunohistochemistry analysis further showed that NK1R was primarily distributed in the intestinal epithelium, lamina propria, submucosa and muscular layer. Administration of SP dramatically increased NK1R expression compared to DSS treatment alone (Figure 10C, D). Immunofluorescence double-staining for NK1R and E-cadherin demonstrated that SP promoted NK1R expression in intestinal epithelial cells (Figure 10E). Consistent with this finding, *in vitro* experiments confirmed that SP elevated NK1R protein expression in TNF-α-stimulated Caco-2 cells, particularly at a dose of 100 nmol (Figure 10F). Subsequently, we investigated whether the inhibitory effects of SP on the cGAS-STING pathway were mediated through activating NK1R. Immunofluorescence analysis defined the colocalization of NK1R and cGAS in Caco-2 cells, providing a fundamental basis for potential correlations between NK1R and the cGAS-STING pathway (Figure 10G). Furthermore, we observed that SP enhanced NK1R expression and markedly suppressed TNF-α-stimulated cGAS signals (Figure 10G). To obtain direct evidence of the interactions between NK1R and the cGAS-STING pathway, Caco-2 cells were pretreated with the NK-1R antagonist (L-732138) at concentrations of 40, 60 and 80 nmol for 2 h prior to SP treatment. Surprisingly, we found that treatment with L-732138 (80 nmol) weakened the ability of SP to inhibit cGAS protein expression (Figure 10H, I). Based on these results, L-732138 at a concentration of 80 nmol for 24 h was applied in subsequent experiments. As hypothesized, L-732138 effectively abrogated the inhibitory effect of SP on the protein expressions of STING, p-TBK1, and p-IRF3 (Figure 10J, K). Subsequently, we addressed whether SP could activate NK1R to diminish inflammation and ferroptosis induced by TNF-α. Interestingly, the effects of SP on inflammatory factors (*IL-8*, *IL-6*, *IL-17A*, and *IFNβ*) were nullified by treatment with L-732138 (Figure 10L-O). Furthermore, L-732138 reversed the inhibitory effects of SP on ferroptosis, elevating *ACSL4* and *PTGS2* mRNA levels while decreasing *FTH1* and *GPX4* mRNA expressions (Figure 10P-S). These results underscore that SP could alleviate inflammation and the ferroptosis process by inhibiting the cGAS-STING pathway in an NK1R-dependent manner.

## Discussion

In this research, we report the role of SP in improving epithelial barrier dysfunction induced by colitis. Our findings not only demonstrate that SP inhibits the cGAS-STING through the suppression of mtDNA release but also elucidate the capability of SP to directly suppress the STING pathway, thereby alleviating inflammation and ferroptosis in the intestinal epithelium during colitis. Furthermore, we verify that the protective effect of SP relies on its receptor NK1R.

In the gastrointestinal tract, SP is derived from immune cells, epithelial cells, and enteric neurons 24, 53. In this study, we demonstrated that endogenous SP was expressed and rapidly increased, particularly in the intestinal epithelium and myenteric neurons during DSS-induced colitis. Koon et al. reported that after DSS removal for an additional 5 days, mice exhibited reduced colitis scores and mild body weight loss, whereas mice treated with the NK-1R antagonist during the recovery phase showed increased colitis clinical scores and further decreased body weight, indicating the therapeutic effects of SP in the recovery phase of colitis 54. We hypothesized that the elevated levels of SP following DSS administration may reflect the organism's self-regulatory and self-defensive mechanism in response to adverse conditions. Although endogenous SP rapidly increases during colitis, it may not be sufficient to adequately protect the intestine from injury. Therefore, supplementation with exogenous SP is required to exert beneficial effects on colitis. The role of SP in organ injury or disease has been controversial. Early-stage research has demonstrated that SP exerts proinflammatory influences on epithelial and immune cells and is involved in inflammatory diseases of the musculoskeletal, respiratory and gastrointestinal systems 55. In contrast, other studies have shown that SP can prevent cholestatic liver injury by regulating inflammatory responses 56. Moreover, SP has been found to promote the recovery of retinal pigment epithelial cells damaged by oxidative stress 57. The data of our study support the beneficial effect of SP on colitis. Supplementation with exogenous SP was able to reverse intestinal barrier disruption, and SP dose-dependently protected against intestinal inflammatory damage. The findings go exactly counter to the idea that elevated levels of SP, observed in the rectum and colon of patients with IBD, correlated with worsened disease activity, while SP deficiency (SP -/-) reduced colon inflammation and ameliorated colitis in mice 17, 58-60. The dual role of SP may be regulated by multiple factors, including clinicopathological conditions and the stages of disease development.

Nevertheless, the potential mechanisms by which SP improves intestinal barrier damage caused by colitis have yet to be explored. In our study, SP suppressed the activation of the cGAS-STING pathway in a dose-dependent manner, both in colitis mice and in TNF-α-induced colonic epithelial cells. Accumulating evidence suggests a connection between the inflammatory response and aberrant activation of the cGAS-STING pathway 61. SP inhibited the expression of inflammatory cytokines and downstream genes of the cGAS-STING pathway in the intestinal epithelium, such as IFNβ, CCL5, and CXCL10. As the epithelial layer is the main form of the colonic barrier, we inferred that SP might reduce intestinal inflammation and promote the repair of the gut mucosal barrier by inactivating the cGAS-STING pathway in intestinal epithelial cells. Similar to SP, atrial natriuretic peptide (ANP), which belongs to the family of bioactive peptides and is secreted by atrial muscle cells, has been shown in a previous study to alleviate colonic inflammation and improve colonic barrier function by inhibiting the STING pathway 62. In addition, andrographolide has been found to decrease inflammatory cytokines in colonic epithelial cells by downregulating the CPT-11-induced cGAS-STING signaling pathway 51.

Importantly, cGAS serves as a cytosolic DNA sensor that detects cytoplasmic DNA and initiates downstream immune responses through the adaptor protein STING 63. A recent study has shown that mtDNA resembling bacterial DNA triggers sterile inflammation and enterocyte injury via the cGAS-STING pathway during intestinal ischemia-reperfusion (I/R) 64. Therefore, further research focusing on the mechanism by which SP modulates the cGAS-STING pathway is essential. Our data suggested that SP possesses the ability to restore DSS-induced damage to mitochondrial structure and function, as well as prevent the loss of MMP in the presence of TNF-α treatment. There is compelling evidence indicating that mitochondrial disruption ultimately results in the leakage of mtDNA from the intestinal epithelial cells in a mouse model of intestinal I/R 42. Here, some gene sequences (mtDNA, COXII, CytB, ND1, and ND2) were applied to measure mtDNA levels. We observed an upregulation of mtDNA levels in the serum of DSS-induced mice or in the culture supernatant of TNF-α-stimulated cells, whereas SP treatment effectively reduced these mtDNA level, thereby preventing mtDNA release. Furthermore, when Caco-2 cells were stimulated with mtDNA, inflammatory factors were induced, and the cGAS-STING pathway was also activated.

Our findings suggested that damaged mtDNA accrued in the cytosol and was released into the extracellular space, where it is sensed by cGAS in intestinal epithelial cells, boosting inflammation in colitis. Notably, the inhibitory effect of SP on the cGAS-STING pathway was reversed upon stimulation with mtDNA. In turn, the absence of mtDNA significantly suppressed TNF-α-induced innate immunity. Our results indicated that SP downregulated the cGAS-STING pathway by inhibiting mtDNA release. Interestingly, even in the absence of mtDNA due to EtBr treatment, SP still exhibited the ability to downregulate the expressions of STING, p-TBK1, p-IRF3, and IFNβ. This suggests the possibility that SP might regulate the STING pathway alternative mechanisms. Surprisingly, we proposed another mechanism by which SP suppresses the cGAS-STING pathway. Protein docking analysis showed that NK1R combined with the protein target STING. To provide more substantial evidence supporting a direct regulatory relationship between NK1R and STING, Caco-2 cells were treated with STING agonists. The results demonstrated that SP significantly inhibited the protein expression of STING, p-TBK1, and p-IRF3 upon DMXAA and SR717 treatment. Thus, we inferred that SP might directly inhibit the STING pathway via activating NK1R in colitis.

It has been reported that ferroptosis is closely related to intestinal barrier dysfunction and the immune system 65. Therefore, it is imperative to investigate the ferroptosis-related biomarkers of intestinal damage and immunotherapy in UC. In addition to triggering inflammatory response, the latest research has revealed that the STING pathway can promote the ferroptosis pathway in septic cardiac dysfunction, pancreatic cancer, and acute kidney injury 52, 66, 67. To elucidate the downstream effectors of cGAS-STING signaling in colitis, we focused on the impact of cGAS-STING signaling on the ferroptosis pathway in colitis. Numerous studies have indicated that inhibiting ferroptosis is an effective approach for alleviating colitis 68, 69. First, we investigated whether SP is involved in the upregulation of ferroptosis in DSS-induced colitis. Our team unveiled that SP reversed the protein expression of ferroptotic markers involving COX-2, FTH1, and GPX4. Similarly, SP significantly increased mRNA levels of *FTH1*, *SLC7A11*, and *GPX4*, but reduced *PTGS2* level. Ferroptosis was closely implicated with the lethal accumulation of ROS. Emerging investigations have proposed that oxidative stress exacerbates ferroptotic cell death in colitis, and is generally recognized as a ferroptosis-related marker 70, 71. Consistent with previous reports, our findings presented evidence of enhanced GSH-Px and T-AOC activity as well as reduced MDA level upon SP treatment, which provided support for the inhibitory effect of SP on the process of ferroptosis. Then, we found mtDNA transfection accelerated the levels of ferroptosis biomarkers and reversed the inhibitory effects of SP on the ferroptosis process in TNF-α-induced Caco-2 cells. In addition, SR717 promoted *PTGS2* and *ACSL4* expression while decreasing *SLC7A11* mRNA level upon TNF-α stimulation. Western blot results also confirmed that SR717 significantly repressed ferroptosis. Hence, we speculated that SP inhibited ferroptosis and inflammation by suppressing mtDNA-cGAS-STING signaling or directly dampening the STING pathway, thereby relieving intestinal damage in colitis.

Lastly, we hypothesized that SP might regulate numerous biological and pathological processes largely through its interaction with its specific receptor. Among the neurokinin receptors, SP has a higher affinity for NK1R compared to NK2R and NK3R 72, 73. NK1R is widely expressed in the intestinal tract and is predominantly localized in the cytomembrane and cytoplasm of many cell types 74, including enteric neurons, interstitial cells of Cajal, epithelial cells, and immune cells 75, 76. In our study, we observed an increased NK1R expression in the colonic epithelium. Importantly, both *in vivo* and *in vitro*, there was an obvious augmentation in NK1R protein expression after SP treatment. Immunofluorescence staining confirmed the colocalization of NK1R with cGAS/STING in Caco-2 cells, indicating that SP activated NK1R while inhibiting the cGAS or STING expression. Furthermore, administration of L-732138 significantly reversed the increase in the expression of STING pathway components, implying that SP suppressed the cGAS-STING pathway by activating NK1R. Notably, we further observed that intervention with L-732138 significantly facilitated the expression of proinflammatory cytokines whereas down-regulated the anti-inflammatory cytokines. Moreover, treatment with L-732138 effectively reversed the suppressive effects of SP on ferroptosis biomarker levels. Thus, we confirmed that SP depended on its receptor NK1R to attenuate inflammation and ferroptosis through the cGAS-STING pathway in colitis. Several limitations of this study should be acknowledged. Further research is needed to explore its clinical potential. Furthermore, there is a lack of effective delivery systems, such as hydrogels and nano-technique-aided. Besides, the precise mechanisms by which the cGAS-STING pathway modulates ferroptosis and the effects of SP on this progress will be investigated in future studies.

## Conclusion

**Figure 11:**
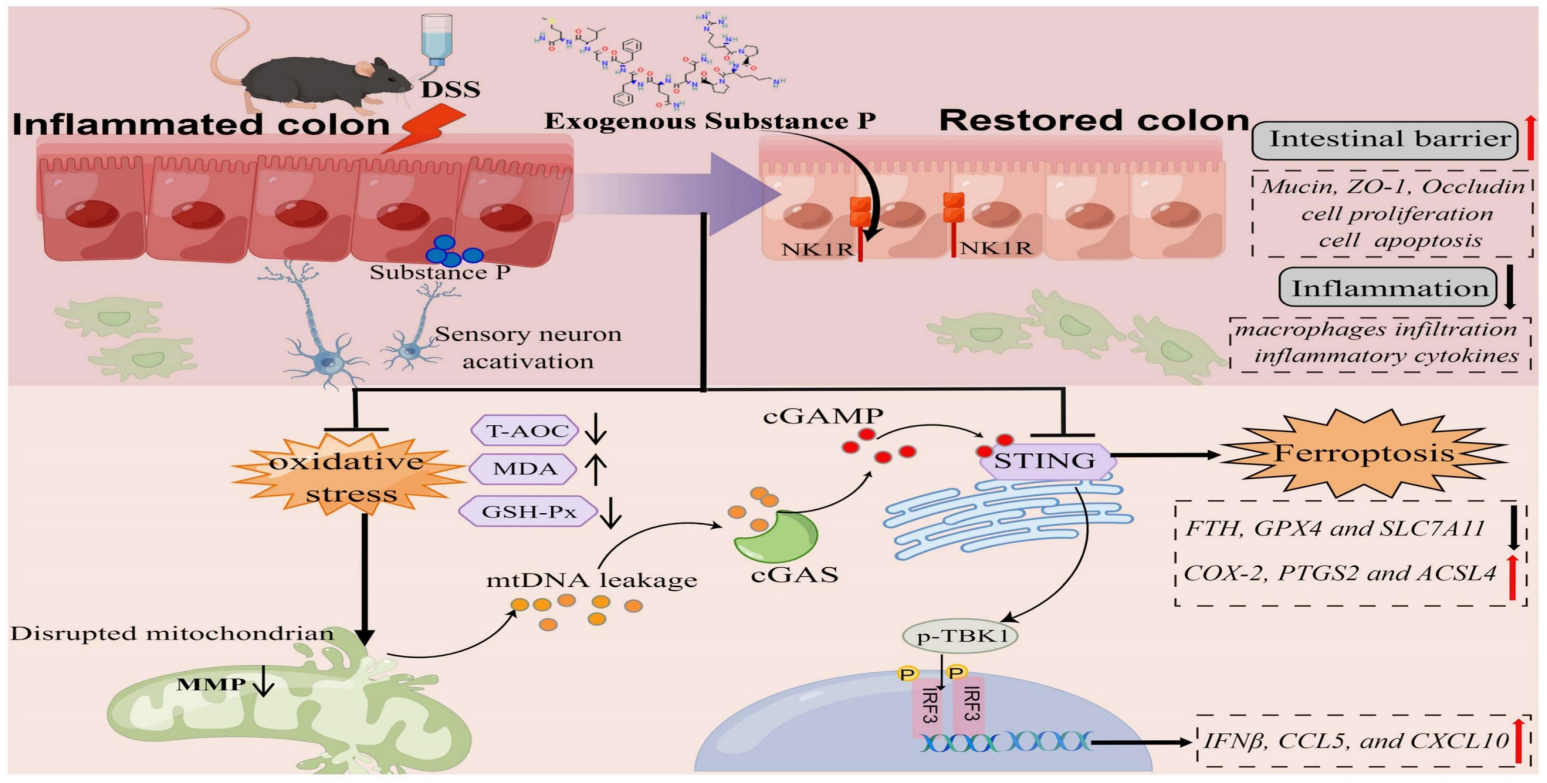
**The schematic diagram explains the mechanism underlying by which SP protects against colitis.** SP improves intestinal mucosal integrity, which was associated with inhibiting mtDNA-cGAS-STING signaling or directly repressing the STING pathway to attenuate inflammatory response and ferroptosis process, alleviating intestinal damage in an NK1R-dependent manner. The figure was drawn by Figdraw.

In summary, SP not only inhibits cGAS-STING via preventing mtDNA leakage into the cytoplasm but also directly suppresses the STING pathway, eventually mitigating inflammation and ferroptosis to alleviate intestinal epithelium damage caused by colitis. Moreover, the protective effects of SP and its underlying action mechanisms rely on its receptor NK1R (Figure 11). To the best of our knowledge, this is the first study to uncover novel mechanisms of SP against colitis, which provides a critical theoretical basis for identifying a promising therapeutic alternative in treating UC and other inflammatory intestinal disorders.

## Supplementary Material

Supplementary figures and tables.

## Abbreviations

SP: substance P
NK1R: neurokinin-1 (NK-1) receptor
cGAS-STING: cyclic GMP-AMP synthase-stimulator of interferon genes
DSS: dextran sodium sulfate
TNF-α: tumor necrosis factor-α
IL-3: interleukin-3
IL-1β: interleukin-1beta
IL-9: interleukin-9
IL-4: interleukin-4
MCP-1: monocyte chemoattractant protein-1
IL-5: interleukin-5
IFN-γ: interferon-gamma
IL-2: interleukin-2
MIP-1α: macrophage inhibitory protein-1 alpha
IL-13: interleukin-13
IL-6: interleukin-6
IL-1α: interleukin-1alpha
G-CSF: granulocyte colony-stimulating factor
Eotaxin: eotaxin-CCL11
MIP-1β: macrophge inhibitory protein-1 beta
IL-12P40: interleukin 12 p40
IL-10: interleukin-10
KC: keratinocyte chemoattractant
IL-17A: interleukin-17
GM-CSF: granulocyte macrophage-colony stimulating factor
IL-12P70: interleukin 12 p70
IL-8: interleukin-8
IL-10: interleukin-10
SOD: superoxide dismutase
GSH-Px: glutathione peroxidase
T-AOC: total antioxidant capacity
MDA: malondialdehyde
p-TBK1: phospho-TANK-binding kinase 1
p-IRF3: phospho-interferon regulatory factor 3
IFNβ: interferon-beta
CXCL10: c-x-c motif chemokine ligand 10
CCL5: chemokine c-c motif ligand 5
GPX4: glutathione peroxidase 4
FTH1: ferritin Heavy Chain 1
COX-2: cyclooxygenase 2
MAP-2: microtubule associated protein 2
PCNA: proliferating cell nuclear antigen
ZO-1: zona occludens 1
SLC7A11: solute carrier family 7 member 11
PTGS2: prostaglandin-endoperoxide synthase 2
ACSL4: acyl-CoA synthetase long-chain family member 4
mtDNA: mitochondrial DNA
18S rRNA: 18S ribosomal RNA
CytB: cytochrome B
COXII: cytochrome c oxidase subunit 2 (COXII)
ND1: NADH dehydrogenase subunits 1
ND2: NADH dehydrogenase subunits 2
BAX: Bcl-2-associated x
BCL-2: B-cell lymphoma-2
