# Supplementary material for: Neuropeptide substance P attenuates colitis by suppressing inflammation and ferroptosis via the cGAS-STING signaling pathway
Source: Int J Biol Sci. 2024 Apr 22;20(7):2507–31. doi: 10.7150/ijbs.94548 (PMC11077368; doi:10.7150/ijbs.94548)
Supplement: Supplementary file 1 — Supplementary figures and tables. [file ijbsv20p2507s1.pdf]

Supplementary Materials for

Neuropeptide substance P attenuates colitis by suppressing inflammation and ferroptosis via the cGAS-STING signaling pathway

This PDF file includes:

Figs. S1 to S5

Other Supplementary Materials for this manuscript include the following:

Table S1 to S2

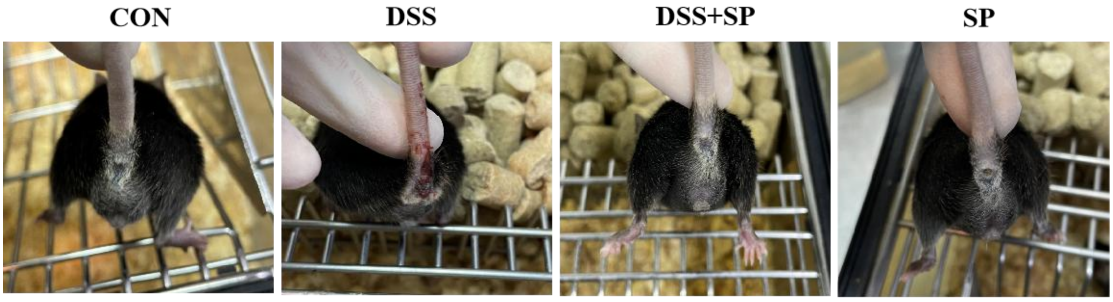

Figure S1 The representative clinical observation and anatomical observation in CON group, DSS group, DSS+SP group, and SP group.

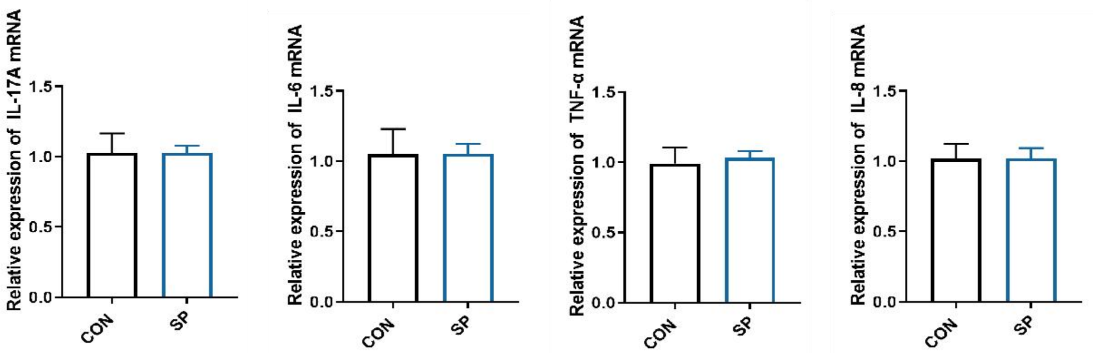

Figure S2 The effects of SP on inflammatory factors in colon tissue. The mRNA levels of IL-17A, IL-6, TNF-α and IL-8 in CON and SP alone group. Data are expressed as the mean ± SEM; n = 10.

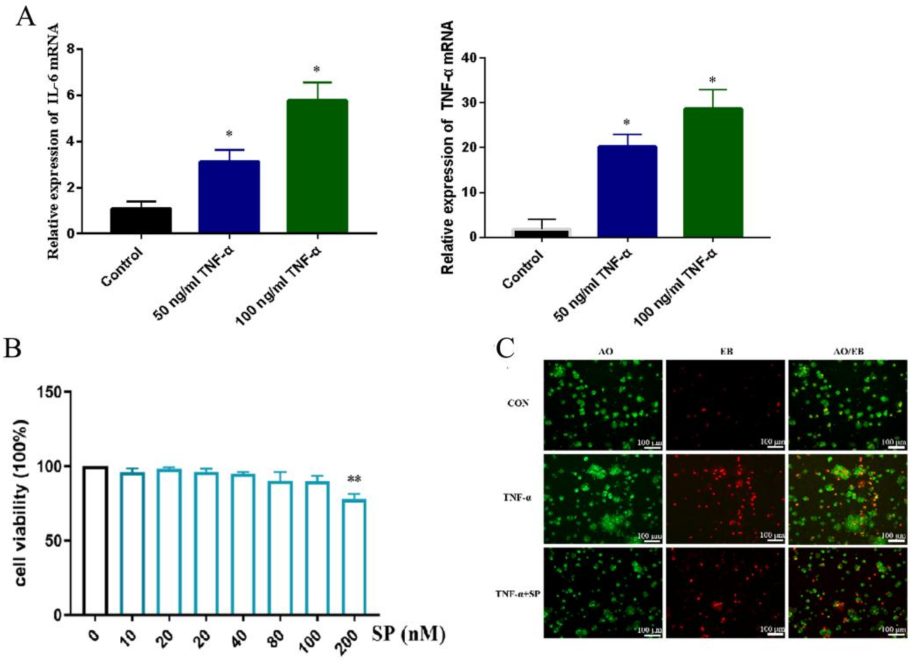

Figure S3 The effect of SP on cell viability and apoptotic cells induced by TNF-α in Caco-2 cells. (A) The mRNA levels of IL-6 and TNF-α in Caco-2 cells treated with TNF-α at the concentration of 50 and 100 ng/ml. (B) The effect of SP on cell viability.

(C) Apoptotic cells were examined by AO/EB staining in Caco-2 cells. Data are expressed as the mean± SEM; n = 4. $^{*}p < 0.05$, $^{**}p < 0.01$ vs. the control group.

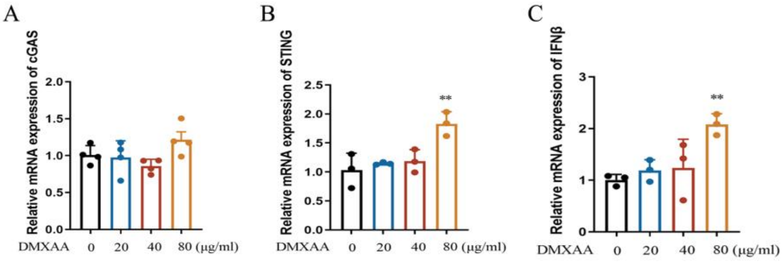

Figure S4 Caco-2 cells were treated with different doses of DMXAA. (A-C) Relative expression of cGAS, STING and IFNβ after DMXAA treatment at the concentration of 20, 40 and 80 μg/mL. Data are expressed as the mean ± SEM; n = 4. $^{**}p < 0.01$ vs. the control group.

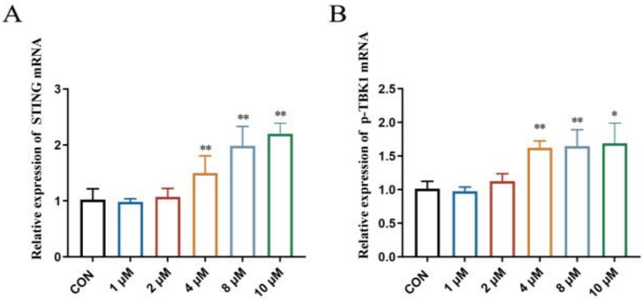

Figure S5 Caco-2 cells were treated with different concentrations of SR717. (A, B) Relative expression of STING and p-TBK1 after SP717 treatment at the concentration of 1, 2, 4, 8 and10 μM. Data are expressed as the mean ± SEM; n = 4. $^{*}p < 0.05$ and $^{**}p < 0.01$ vs. the control group.

Table S1 Sequence information of primers for mice colon tissue

Gene (mice)	Forward Primer (5' - 3 ')	Reverse Primer (5' - 3')
SP	GCGACCAGATAAAGGAGGAGCTGC	CTGCTGAGGCTTGGGTCTCCG
IL-1β	GCTGCTTCCAAACCTTTGAC	AGCTTCTCCACAGCCACAAT
IL-6	TAGTCCTTCCTACCCCAATTTCC	TTGGTCCTTAGCCACTCCTTC
IL-17A	CACAGCCTCAGAGTCCTTCAT	ACCTTCCGCATTAGCACAGA
TNF-α	CCGATGGGTTGTACCTTGTC	AGATAGCAAATCGGCTGACG
IL-8	GCAGAGGGTTGTGGAGAAGT	CCCTACAACAGACCCACACA
IL-10	ACTGGCTGTTGCCTTCTTGG	TGGGGTGGAAAGGTGTGGAA
IL-4	CCCCAGCTAGTTGTCATCCTG	CAAGTGATTTTTGTCGCATCCG
ZO-1	CCACCTCTGTCCAGCTCTTC	CACCGGAGTGATGGTTTTCT
Claudin-2	GTCATCGCCCATCAGAAGAT	ACTGTTGGACAGGGAACCAG
Occludin	ATGTCCGGCCGATGCTCTC	TTTGGCTGCTCTTGGGTCTGTAT
cGAS	ATGTTTAAACTGGAAGTCCCC	AAGAAGTGTTACAGCAGGGCT
STING	GGTCACCGCTCCAAATATGTAG	CAGTAGTCCAAGTTCGTGCGA
p-IRF3	CGAAGTGGGAGACAGGACGC	GAGCAGCTCCTCGCTCACTG
IFNβ	TGGGTGGAATGAGACTATTGTTGTACG	CAAGTGGAGAGCAGTTGAGGACATC
CXCL10	AAGTGCTGCCGTCATTTTCT	GTGGCAATGATCTCAACACG
CCL5	CCTCTATCCTAGCTCATCTCCA	ATTTCTGCCTCATCCTGCT
GPX4	TTCTCAGCCAAGGACATCG	CACTCAGCATATCGGGCAT
FTH1	GCCGAGAAACTGATGAAGCTGC	GCACACTCCATTGCATTCAGCC
SLC7A11	GATGCTGTGCTTGGTCTTGA	GCCTACCATGAGCAGCTTTC
PTGS2	AAATGCTGGTGTGGAAGGT	TTGTTGCTCTAGGCTTTGCT
mtDNA	CTAGCAGAAACAAACCGGGC	CCGGCTGCGTATTCTACGTT
18S rRNA	CCGCCGCCATGTCTCTAGT	CTTTCCTCAACACCACATGAGC
mtCytb1	AATCCACTAAACACCCCACCC	GCTTCGTTGCTTTGAGGTATGA
mtCOXII	ATAACCGAGTCGTTCTGCCAAT	TTTCAGAGCATTGGCCATAGAA
mtND1	GGATCCGAGCATCTTATCCA	GGTGGTACTCCCTCTGTAAA
GAPDH	TTCCTACCCCCAATGTATCCG	CATGAGGTCCACCACCCTGTT

Table S2 Sequence information of primers for Caco-2 cells

Gene (Human).	Forward Primer (5' - 3 ')	Reverse Primer (5' - 3')
IL-6	CATCACTGGTCTTTTGGAG	TGGATCAGGACTTTTGTAC
IL-17A	AATCTCCACCGCAATGAGGA	ACGTTCCCATCAGCGTTGA
TNF-α	TCTCTCAGCTCCACGCCATT	CCCAGGCAGTCAGATCATCTTC
IL-1β	AAACAGATGAAGTGCTCCTTCCAGG	TGGAGAACACCACTTGTTGCTCCA
IL-8	AGGAACCATCTCACTGTGTG	ATTTGGGGTGGAAAGGTT
cGAS	AAGGATAGCCGCCATGTTTCT	TGGCTTTCAGCAAAAGTTAGG
STING	GCGGCTGTATATTCTCCTCCCATTG	TGCTGTTGCTGTAAACCCGATCC
p-IRF3	CTGGGGCCCTTCATTGTAGAT	GGCACAACCTTGACCATCAC
IFNβ	TGTCGCCTACTACCTGTTGTGC	AACTGCAACCTTTCGAAGCC
CXCL10	CTTCCAAGGATGGACCACAC	AGCAGGGTCAGAACATCCAC
CCL5	CCAGCAGTCGTCTTTGTCAC	CTCTGGGTTGGCACACACTT
mtND1	GCTACGACCAACTCATAC	GAATGCTGGAGATTGTAATG
mtND2	CACAGAAGCTGCCATCAAGTA	CCGGAGAGTATATTGTTGAAGAG
mtCOXII	ATGACCCACCAATCACATGC	ATCACATGGCTAGGCCGGAG
COX-2	CGGTGAAACTCTGGCTAGACAG	GCAAACCGTAGATGCTCAGGGA
PTGS2	CCTGTGCCTGATGATTGC	CTGATGCGTGAAGTGCTG
SLC7A11	CCCAGATATGCATCGTCCTT	CCTGGGTTTCTTGTCCCATA
ACSL4	GCTTCCTATCTGATTACCAGTGTTGA	GTCCACATAAATGATATGTTTAACACAACT
GPX4	AGATCCACGAATGTCCCAAG	CCTCCTCCTTAAACGCACAC
FTH1	CCATCAACCGCCAGATCAACCTG	GTTTCTCAGCATGTTCCCTCTCCTC
GAPDH	TGGAGCTGCAGAGGATGATTG	CCCAGTTGAAGTTGCCGTCAG
